# Oncogenic KRAS triggers MAPK-dependent errors in mitosis and MYC-dependent sensitivity to anti-mitotic agents

**DOI:** 10.1038/srep29741

**Published:** 2016-07-14

**Authors:** David Perera, Ashok R. Venkitaraman

**Affiliations:** 1 The Medical Research Council Cancer Unit, University of Cambridge, Cambridge, CB2 0XZ, United Kingdom

## Abstract

Oncogenic KRAS induces cell proliferation and transformation, but little is known about its effects on cell division. Functional genetic screens have recently revealed that cancer cell lines expressing oncogenic KRAS are sensitive to interference with mitosis, but neither the mechanism nor the uniformity of anti-mitotic drug sensitivity connected with mutant KRAS expression are yet clear. Here, we report that acute expression of oncogenic KRAS in HeLa cells induces mitotic delay and defects in chromosome segregation through mitogen-activated protein kinase (MAPK) pathway activation and de-regulated expression of several mitosis-related genes. These anomalies are accompanied by increased sensitivity to anti-mitotic agents, a phenotype dependent on the transcription factor MYC and its downstream target anti-apoptotic protein BCL-XL. Unexpectedly, we find no correlation between *KRAS* mutational status or *MYC* expression levels and anti-mitotic drug sensitivity when surveying a large database of anti-cancer drug responses. However, we report that the co-existence of *KRAS* mutations and high *MYC* expression predicts anti-mitotic drug sensitivity. Our findings reveal a novel function of oncogenic KRAS in regulating accurate mitotic progression and suggest new avenues to therapeutically target *KRAS*-mutant tumours and stratify patients in ongoing clinical trials of anti-mitotic drugs.

Recent studies implicate several proteins controlling mitotic progression as being synthetic lethal with oncogenic KRAS^G13D^ in human colorectal cancer cells^1^,^2^,^3^, consistent with the heightened sensitivity of these cells to paclitaxel when compared to their wild-type counterparts^2^. However, neither the mechanism nor the uniformity of anti-mitotic drug sensitivity connected with mutant KRAS expression is yet clear. Indeed, several lines of evidence suggest that mutant KRAS expression *per se* may not be a marker of anti-mitotic drug sensitivity. For example, inhibition of the mitogen-activated protein kinase (MAPK) pathway, which is hyper-activated by oncogenic KRAS, sensitises cancer cells to the microtubule stabiliser paclitaxel (also known as Taxol)^4^,^5^,^6^. Moreover, lung cancer cell lines harbouring *KRAS* mutations are significantly more resistant than cell lines with wild-type *KRAS* to growth inhibition induced by the anti-mitotic agent GSK923295, an inhibitor of the kinesin centromere-associated protein E (CENP-E)^7^. Therefore, it remains unclear if or how mutations activating KRAS may confer sensitivity to anti-mitotic chemotherapeutics.

One hypothesis is that oncogenic KRAS induces poorly characterised mitotic alterations, termed ‘mitotic stress’, that underlie tumour sensitivity to anti-mitotic agents^2^. Consistent with this notion, pancreatic ductal adenocarcinomas, >90% of which harbour mutant forms of *KRAS*^8^, frequently exhibit abnormal mitotic figures and aneuploid chromosome number^9^,^10^. But whether these anomalies are direct consequences of KRAS activation has not yet been determined. Indeed, although *KRAS* is the *RAS* family member most often mutated in human cancer^11^, it is another *RAS* gene, *HRAS*, which is better studied in this regard. Thus, over-expression of oncogenic HRAS^G12V^ is reported to provoke several mitotic defects including centrosome amplification, micronuclei formation, chromosome mis-alignment and weakening of the spindle assembly checkpoint^12^,^13^,^14^,^15^,^16^,^17^, for which no mechanistic explanation has yet been elucidated. Whether similar anomalies occur in cells expressing mutant KRAS, and if so, through what mechanism, remain open questions.

We have investigated these issues, and report here that oncogenic KRAS provokes errors in mitotic chromosome alignment and segregation dependent on activation of MAPK signalling. Contrary to expectation, however, *KRAS* mutation status does not correlate with increased sensitivity to anti-mitotic agents when analysing a small in-house panel of cancer cell lines, three isogenic cell line pairs or a large database of anti-cancer drug responses. Instead, and consistent with a recent report^18^, we identify an apoptotic mechanism regulated by the transcription factor MYC that determines the sensitivity of *KRAS*-mutant cells to anti-mitotic drugs, and show that, consistent with this mechanism, the co-existence of *KRAS* mutations with elevated *MYC* expression predicts sensitivity to anti-mitotic drugs. Our findings open new avenues for therapeutic intervention in *KRAS*-mutant cancers, highlighted by the increasing clinical acceptance of therapeutic regimes combining anti-mitotic drugs with other agents for *KRAS*-mutant lung or pancreatic cancers.

## Results

### Oncogenic KRAS expression provokes defective progression through mitosis in a MAPK-dependent manner

**Figure 1:**
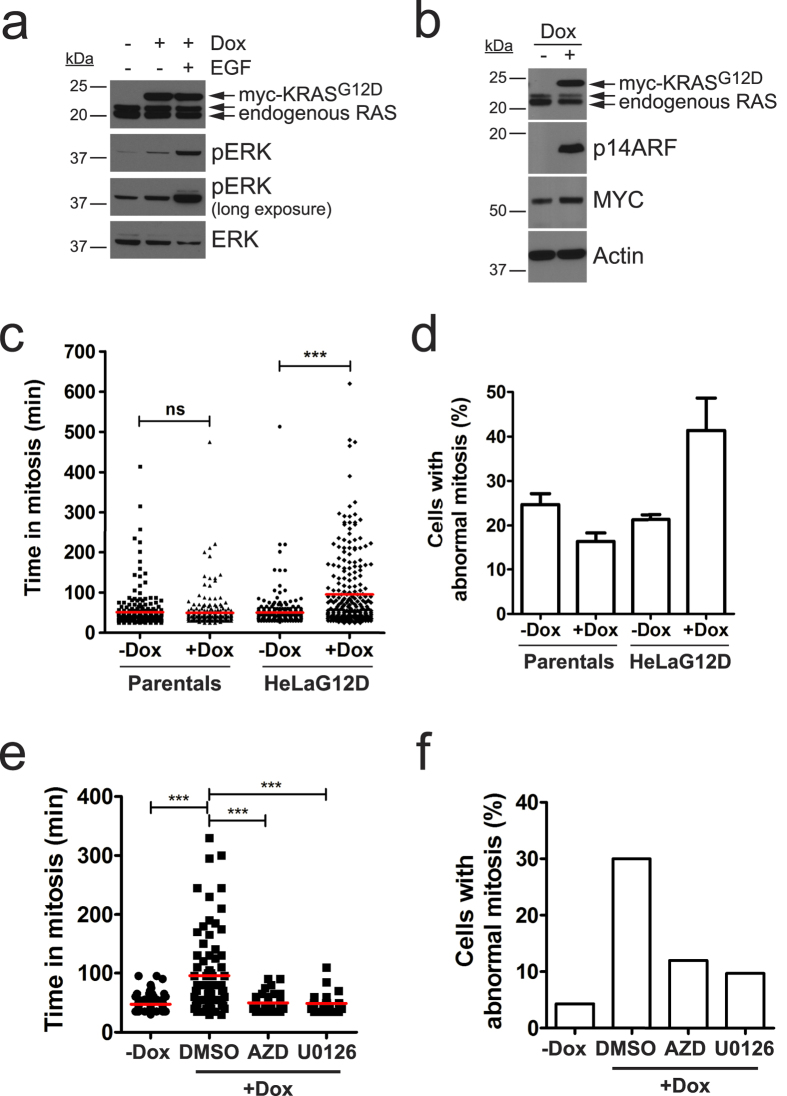
Expression of KRAS^G12D^ in HeLa cells leads to mitotic defects in a MAPK-dependent manner. (**a**) HeLaG12D cells were treated with doxycycline (Dox) for 24 hours, and 10 ng/ml EGF was added 5 minutes before harvesting. Equal amounts of protein lysates were blotted with the indicated antibodies. Top arrow points to ectopic myc-tagged KRAS^G12D^, while bottom 2 arrows point to endogenous RAS isoforms. (**b**) HeLaG12D cells were treated with doxycycline for 5 days, protein lysates were prepared and blotted with the indicated antibodies. Actin was used as a loading control. (**c**) HeLaG12D cells expressing GFP-H2B were treated with doxycycline for 24 hours then monitored by time-lapse microscopy for a further 24 hours. The scatter dot plot shows time spent in mitosis (scored as the time taken from NEB to anaphase onset), represented in minutes. HeLa FRT/TO parental cells were used as controls. Data was obtained from 3 independent experiments, and >250 cells were analysed for each condition. Red lines represent mean values. *ns*, not significant; ***p < 0.0001 (Mann Whitney test). (**d**) Bar graph depicting the percentage of cells with abnormal division (including defects in chromosome alignment and/or segregation, as well as multipolar divisions) from the time-lapse movies in (**c**). Bars represent mean values ± S.E.M. from 3 independent experiments. (**e**) GFP-H2B-expressing HeLaG12D cells were treated with doxycycline for 24 hours, then 0.5 μM AZD6244 (AZD) or 10 μM U0126 were added before monitoring by time-lapse microscopy. Scatter dot plot shows time spent in mitosis as in (**c**). Data was obtained from 2 independent experiments. Red lines represent mean values. ***p < 0.0001 (Mann Whitney test). (**f**) Bar graph depicting the percentage of cells with abnormal division from the time-lapse movies in (**e**).

Previous reports suggest *KRAS*-mutant cancer cells display abnormalities during mitosis^2^. To explore this, we generated a stable cell line where expression of KRAS^G12D^ can be induced by treatment with doxycycline (Dox). Figure 1a shows expression of ectopic KRAS^G12D^ after a 24-hour treatment of HeLa FRT/TO KRAS^G12D^ cells (from here on, termed HeLaG12D) with doxycycline. The transgene has a single myc tag at its amino terminus, distinguishing it in size from the endogenous RAS proteins. As expected, activation of the downstream MAPK pathway occurs upon doxycycline treatment, as detected by increased phosphorylation and nuclear translocation of Extracellular signal-regulated kinase (ERK) 1 and 2 (Fig. 1a and Supplementary Fig. S1). As a positive control, we treated HeLaG12D cells with Extracellular Growth Factor (EGF), which triggers a considerably higher increase in phosphorylated ERK1/2. Known downstream effects of RAS-MAPK pathway activation such as elevated expression and/or stabilization of MYC and tumour suppressor ARF are also observed at later time points (5 days post-doxycycline; Fig. 1b), demonstrating that this model system recapitulates several characteristics of RAS pathway activation.

In order to analyse mitotic progression and chromosome behaviour shortly after expression of KRAS^G12D^, we stably integrated GFP-tagged histone H2B into HeLaG12D cells, and performed time-lapse imaging 24 h after addition of doxycycline. As shown in Fig. 1c, untreated cells spent an average of 50 minutes in mitosis (scored from nuclear envelope breakdown [NEB] to anaphase; n = 250 cells), while a significant proportion of doxycycline-treated cells showed delayed progression through mitosis (mean value of 95 minutes; n = 252 cells). Closer inspection of the time-lapse data revealed that KRAS^G12D^-expressing HeLa cells displayed a variety of mitotic defects including errors in chromosome alignment and segregation (Fig. 1d and Supplementary Fig. S2). We note that, in most cases, these defects were relatively mild, with 1–2 unaligned or lagging chromosomes in anaphase, consistent with the fact that KRAS is not a direct mediator of mitosis, and instead suggesting that oncogenic KRAS may exert subtle regulatory effects on the mitotic machinery. Importantly, neither parental cells devoid of transgene expression nor cells expressing wild-type KRAS showed mitotic delay or defects in chromosome alignment or segregation upon doxycycline treatment (Fig. 1c,d and Supplementary Fig. S3). Taken together, these results indicate that acute expression of oncogenic KRAS triggers aberrant mitotic division associated with delays in progression through mitosis.

In order to explore the mechanism by which KRAS^G12D^ leads to defects in mitosis, we treated H2B-GFP-expressing HeLaG12D cells with doxycycline for 24 h, then added two different MEK1/2 inhibitors: the first-generation compound, U0126, and the highly potent and selective inhibitor, AZD6244. Treatment with either inhibitor prevented mitotic delay as well as the appearance of mitotic defects triggered by KRAS^G12D^ expression (Fig. 1e,f). This result suggests that mitotic abnormalities elicited by oncogenic KRAS expression require MAPK pathway activation.

To further analyse the mitotic defects in HeLaG12D cells, we looked at the ability of KRAS^G12D^-expressing cells to form a stable bipolar spindle upon release from a Monastrol block^19^,^20^. Interestingly, the majority of doxycycline-treated HeLaG12D cells exhibited metaphase spindles with abnormal geometry upon Monastrol washout, with poles often out of line with normal spindle orientation (Supplementary Fig. S4a–c). Spindles also appeared longer and narrower than in control, non-induced cells (Supplementary Fig. S4d,e). These results suggest expression of KRAS^G12D^ influences one or more processes essential for mitotic progression. In fact, quantitative PCR analysis showed expression of several mitosis-related genes was down-regulated in doxycycline-treated HeLaG12D cells prior to mitotic entry (Supplementary Fig. S5), providing a potential explanation for the observed mitotic defects in these cells. The list of de-regulated mitotic genes includes centromere protein A (*CENPA*) and centromere protein M (*CENPM*), which encode proteins essential for the assembly of a functional kinetochore^21^, cohesin associated factor *PDS5B*^22^, and several genes encoding proteins involved in mitotic spindle formation and/or maintenance, such as microtubule-associated protein 7 (*MAP7*, also known as *Ensconsin* or *E-MAP*-*115*)^23^,^24^, large tumour suppressor kinase 2 (*LATS2*)^25^ and kinesin family member 2C (*KIF2C*, also known as *MCAK*)^26^,^27^.

**Figure 2:**
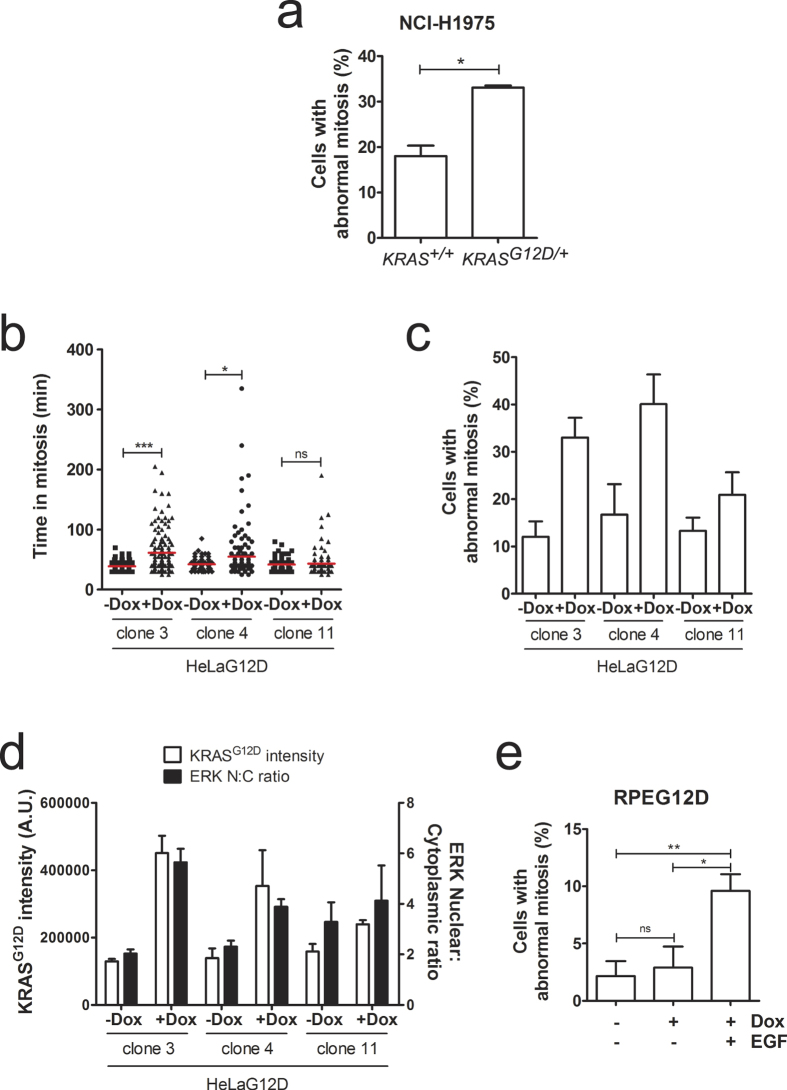
KRAS^G12D^-induced mitotic defects are accompanied by increased nuclear accumulation of ERK1/2. (**a**) Asynchronously growing GFP-H2B-expressing NCI-H1975 *KRAS*^+/+^ and *KRAS*^*G12D*/+^ cells were monitored by time-lapse microscopy for 24 hours. Bar graph depicts the percentage of cells with abnormal division (mean ± S.E.M. from 3 independent experiments), with >40 cells analysed per experiment for each cell line. *p < 0.05 (paired t-test). (**b**) Individual HeLaG12D clones expressing GFP-H2B were treated with doxycycline for 24 hours then monitored by time-lapse microscopy for a further 24 hours. The scatter dot plot shows time spent in mitosis, represented in minutes. Data was obtained from 3 independent experiments, and 67–97 cells were analysed for each condition. Red lines represent mean values. *ns*, not significant; *p < 0.05; ***p < 0.0001 (Mann Whitney test). (**c**) Bar graph depicting the percentage of cells with abnormal division from the time-lapse movies in (**b**). Bars represent mean values ± S.E.M. from 3 independent experiments. (**d**) Individual HeLaG12D clones were treated with doxycycline for 24 hours, then fixed and stained for the myc tag (KRAS^G12D^) and ERK1/2. Bar graph depicts KRAS^G12D^ pixel intensity (left Y axis) and nucleo-cytoplasmic (N:C) ratio of ERK1/2 (right Y axis). (**e**) GFP-H2B-expressing RPEG12D cells were treated with doxycycline and synchronised in G1/S by addition of thymidine, then released from the G1/S block in the presence or absence of EGF and monitored by time-lapse microscopy. Bar graph depicts the percentage of cells with abnormal division. Bars represent mean values ± S.E.M. from 3 independent experiments, with 16–64 cells analysed per condition in each experiment. *ns*, not significant; *p < 0.05; **p < 0.005 (paired t-test).

We then decided to check whether expression of KRAS^G12D^ provokes mitotic defects in a different cell line. For this purpose, we stably integrated GFP-tagged histone H2B into an isogenic pair of lung cancer cell lines that only differ in their *KRAS* genotype (NCI-H1975 *KRAS*^+/+^ and *KRAS*^*G12D*/+^), and monitored cell division by time-lapse microscopy. As shown in Fig. 2a, GFP-H2B-expressing *KRAS*^*G12D*/+^ cells displayed a higher incidence of chromosome alignment and segregation defects compared to the parental NCI-H1975 *KRAS*^+/+^ cells, suggesting KRAS^G12D^-triggered mitotic defects are not a phenotype exclusive of HeLa cells. We then engineered immortalised, non-transformed retinal pigment epithelial cells (hTERT-RPE-1; hereafter referred as RPE) to express KRAS^G12D^ upon doxycycline treatment. Interestingly, doxycycline-treated RPEG12D cells did not display errors in mitosis or a delay in mitotic progression when compared to un-induced RPEG12D cells (Supplementary Fig. S6a,b). We noticed however that expression of ectopic KRAS^G12D^ protein was lower in the polyclonal RPEG12D population than in HeLaG12D cells (data not shown), prompting us to investigate whether the increased expression levels of KRAS^G12D^ were responsible for the induction of mitotic defects. To address this possibility, we first monitored mitotic progression in 3 independent HeLaG12D clones. As shown in Fig. 2b,c, mitotic defects and delay were only observed in clones 3 and 4, which show higher expression of KRAS^G12D^ than clone 11 (Supplementary Fig. S6c). This result suggests that elevated levels of KRAS^G12D^ expression may be required for the induction of errors in progression through mitosis. We then analysed mitotic progression in RPEG12D clone 4, which expresses KRAS^G12D^ at levels comparable to the HeLaG12D polyclonal population (Supplementary Fig. S6c). Surprisingly, no mitotic defects were observed in these cells either (Supplementary Fig. S6d,e), suggesting elevated expression of oncogenic KRAS *per se* is insufficient to trigger defective cell division.

Interestingly, we noticed that RPEG12D cells were unable to fully activate the MAPK pathway, as measured by their inability to induce translocation or ERK1/2 into the nucleus upon doxycycline treatment (Supplementary Fig. S6f and data not shown). However, translocation of ERK1/2 into the nucleus was readily apparent in HeLaG12D cells, both in the polyclonal population and in individual clones 3 and 4 (Fig. 2d and Supplementary Fig. S1). These results suggest that the inability of KRAS^G12D^ to fully activate MAPK pathway in RPE cells might be responsible for the lack of induction of mitotic defects in these cells, consistent with the fact that KRAS^G12D^-triggered mitotic errors in HeLa cells are mediated by MEK (see Fig. 1). To test this hypothesis, we treated RPEG12D cells with EGF, which induces hyper-activation of the MAPK pathway independently of oncogenic KRAS activation. Strikingly, EGF-treated RPEG12D cells showed an increase in the incidence of mitotic errors (Fig. 2e). Taken together, our results suggest that abnormal activation of the MAPK pathway, which occurs in most cell types following oncogenic KRAS activation, results in defective mitotic progression.

### Oncogenic KRAS expression increases sensitivity to anti-mitotic drugs

**Figure 3:**
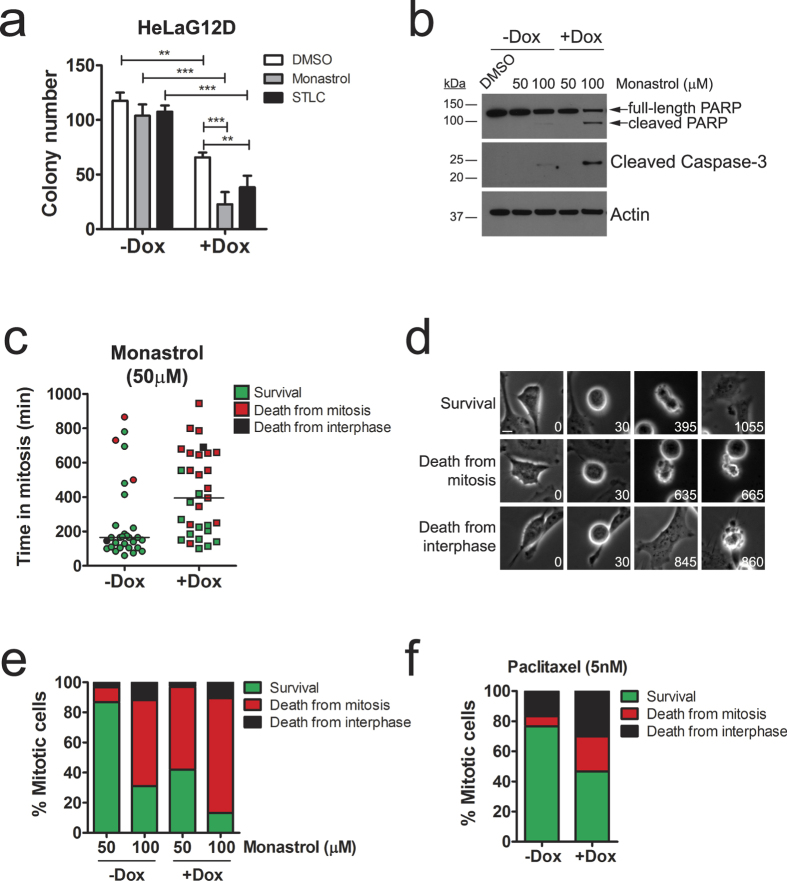
KRAS^G12D^ expression increases sensitivity to anti-mitotic drugs in HeLa cells. (**a**) Bar graph depicting the number of colonies in HeLaG12D cells pre-treated with doxycycline for 48 hours, then treated with 100 μM Monastrol or 5 μM STLC for 3 days and cultured in the absence of drugs for a further 5 days. Bars represent mean values ± S.E.M. (n = 3 independent experiments). **p < 0.01; ***p < 0.001 (Two-way ANOVA with Bonferroni post-tests). (**b**) Immunoblots of HeLaG12D cells treated with doxycycline for 48 hours, then with Monastrol for a further 24 hours. Protein lysates were probed for PARP and cleaved (i.e. active) Caspase-3. Actin was used as a loading control. (**c**) HeLaG12D cells were pre-treated with doxycycline then incubated with Monastrol and filmed by phase-contrast time-lapse microscopy. Scatter dot plot shows time from mitotic entry (NEB) to either mitotic exit or death (whatever comes first). Dot colors reflect the fate of individual cells: green (mitotic exit and survival of daughter cells, at least up to the point the filming ends); red (death from mitosis); black (mitotic exit and death from interphase). Only cells entering mitosis were analysed (n ≥ 30). Horizontal bars represent mean values. (**d**) Representative images from time-lapse movies analysed in (**c**), depicting the three different fates scored. Note that treatment with this concentration of Monastrol led to some apparently normal divisions and 2 (sometimes more) daughter cells. Numbers represent time in minutes from NEB. Scale bar, 10 μm. (**e**) Stacked bar graph representing the fate of HeLaG12D cells pre-treated with doxycycline, then challenged with two different concentrations of Monastrol. Bar colors represent cell fates as described in (**c**,**d**). (**f**) Stacked bar graph representing the fate of HeLaG12D cells pre-treated with doxycycline, then challenged with 5 nM paclitaxel and monitored by phase-contrast time-lapse microscopy. Bar colors represent cell fates as described in (**c**,**d**).

We hypothesized that the mitotic delay observed in HeLaG12D cells could enhance sensitivity to treatments perturbing normal mitotic progression. Indeed, the colony-forming ability of doxycycline-treated HeLaG12D cells was reduced compared to untreated cells when cultured in the presence of the kinesin Eg5 inhibitors Monastrol or S-trityl-L-cysteine (STLC; Fig. 3a). As a control, we treated HeLa parental cells with doxycycline, then with Eg5 inhibitors. As shown in Supplementary Fig. S7a, no differences in colony-forming ability were observed in doxycycline-treated vs. untreated parental cells challenged with Eg5 inhibitors. Importantly, expression of KRAS^G12D^ on its own sufficed to reduce colony formation (Fig. 3a, white bars, DMSO), indicating KRAS^G12D^ expression has anti-proliferative effects in HeLa cells and consistent with the fact that it compromises mitotic cell division. Indeed, when doxycycline-treated HeLaG12D cells were followed by time-lapse microscopy over a period of 2 days after release from a double thymidine block, it was apparent that abnormal divisions induced by KRAS^G12D^ expression led to a higher incidence of death of ‘grand-daughter’ cells (Supplementary Table S1). This finding is consistent with the decreased ability of these cells to form colonies in the long term.

Interestingly, we also observed signs of apoptosis (as determined by cleavage of caspase-3 and PARP) as early as 24 h after Eg5 inhibition in doxycycline-treated vs. untreated HeLaG12D cells (Fig. 3b and Supplementary Fig. S7b), indicating the combination of anti-mitotic drug treatment with acute KRAS^G12D^ expression leads to premature cell death. Similar results were observed when challenging cells with paclitaxel (Supplementary Fig. S7c). To investigate this phenotype in more detail, we performed phase-contrast time-lapse imaging of doxycycline-treated HeLaG12D cells upon addition of 50 μM Monastrol, half the concentration required for maximal Eg5 inhibition^20^. As shown in Fig. 3c, control HeLaG12D cells (-doxycycline) were only partially delayed in mitosis (median value = 180 minutes) and the majority of cells stayed alive during the period of filming (48 hours). In contrast, Monastrol treatment led to a significant delay in mitosis in doxycycline-treated HeLaG12D cells, with 50% of cells dying during this protracted mitotic arrest (Fig. 3c,d). At higher concentrations of Monastrol (100 μM), the difference between doxycycline-treated and untreated HeLaG12D cells was still apparent albeit less significant (80% cells dying during mitosis +doxycycline, compared to 50% -doxycycline; Fig. 3e). Similar results were observed with a different Eg5 inhibitor (STLC) and with a different spindle poison (paclitaxel; Fig. 3f and Supplementary Fig. S7d). Of note, low concentrations of paclitaxel (5–10 nM), comparable to the intracellular level of paclitaxel in treated patients and cell lines^28^, were required to expose different sensitivity between doxycycline-treated and untreated HeLaG12D cells (Fig. 3f and Supplementary Fig. S7c,e). At these concentrations, the mitotic delay was mild (median value = 107 minutes -doxycycline, 130 minutes +doxycycline; n = 30 cells analysed per condition; Supplementary Fig. S7e, left panel). At higher concentrations (30–100 nM), the delay in mitosis was more pronounced and, although all cells died in mitosis irrespective of doxycycline exposure, KRAS^G12D^-expressing HeLa cells (+Dox) died significantly faster than non-expressing cells (-Dox; Supplementary Fig. S7e, middle and right panels), suggesting KRAS^G12D^ expression primes cells for the induction of apoptosis, leading to faster cell death upon anti-mitotic drug treatment. Taken together, these results suggest expression of KRAS^G12D^ can lead to increased sensitivity to anti-mitotic agents by two mechanisms: by lowering the death threshold prior to mitotic entry, and by delaying mitotic progression, which allows for death signals to accumulate.

### Transcriptional down-regulation of *BCL-XL* by MYC mediates cell death in response to anti-mitotic treatments in KRAS^G12D^-expressing cells

**Figure 4:**
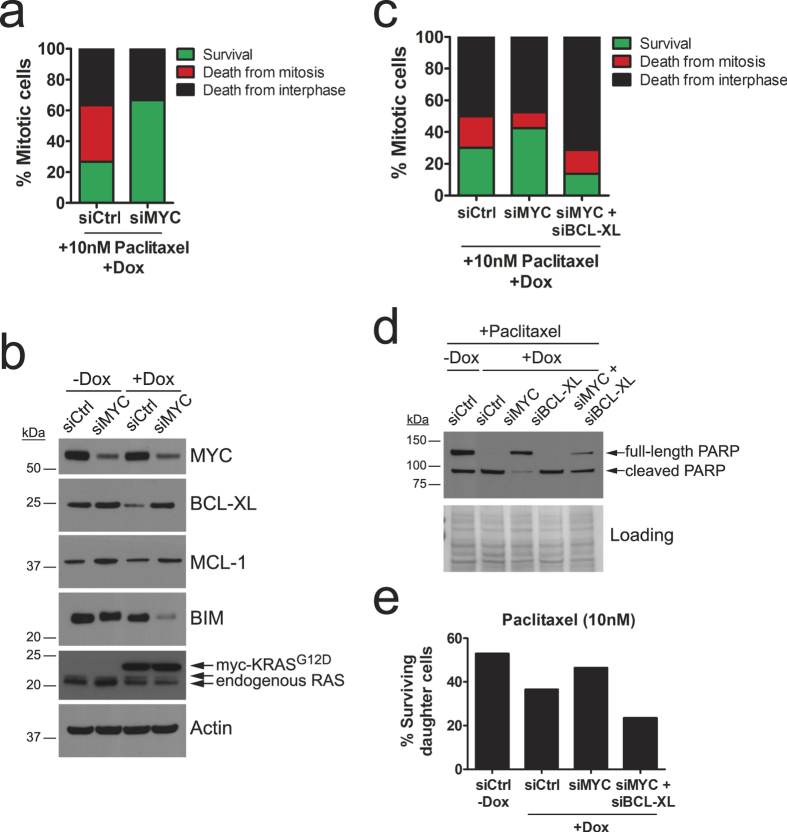
Cell death elicited by anti-mitotic agents in KRAS^G12D^-expressing HeLa cells is mediated by MYC and BCL-XL. (**a**) Stacked bar graph representing the fate of HeLaG12D cells pre-treated with doxycycline, transfected with MYC or control siRNA oligos, then challenged with 10 nM paclitaxel and monitored by phase-contrast time-lapse microscopy (n = 30 cells analysed per condition). Bar colors represent cell fates as described in Fig. 3c. (**b**) Immunoblot analysis of HeLaG12D cells untreated or treated with doxycycline for 48 hours, then transfected with control or MYC siRNA oligos and probed with the indicated antibodies. Actin was used as a loading control. (**c**) Stacked bar graph representing cell fate of HeLaG12D cells pre-treated with doxycycline, transfected with control, MYC and BCL-XL siRNA oligos, then challenged with 10 nM paclitaxel and monitored by phase-contrast time-lapse microscopy (n = 80 cells analysed per condition in two independent experiments). Bar colors represent cell fates as described in Fig. 3c. (**d**) Immunoblots of HeLaG12D cells pre-treated with doxycycline, transfected with control, MYC and/or BCL-XL siRNA oligos, then challenged with 100 nM paclitaxel for 28 hours. PARP cleavage was monitored as a sign of apoptotic cell death. Equal amounts of protein lysates were loaded for each sample, and Ponceau staining was used as a loading control, as Actin levels decreased in samples with significant levels of death. (**e**) HeLaG12D cells were pre-treated with doxycycline, transfected with control, MYC and BCL-XL siRNA oligos, then challenged with 10 nM paclitaxel and monitored by phase-contrast time-lapse microscopy. Bar graph depicts the percentage of daughter cells surviving the 48-hour filming period.

To explore the mechanism of cell death of KRAS^G12D^-expressing cells after exposure to anti-mitotic chemotherapeutics, we focused on the well-established connection between oncogenic RAS and MYC. Expression of oncogenic RAS leads to up-regulation of the transcription factor MYC via ERK-mediated phosphorylation and subsequent protein stabilisation^29^,^30^. As described above (see Fig. 1b), HeLaG12D cells display increased expression levels of MYC, confirming this observation. MYC is known to regulate transcription of several members of the BCL-2 protein family, key mediators of apoptotic cell death, in several contexts^31^,^32^,^33^,^34^. Furthermore, MYC has been recently shown to be a key mediator of cell death in response to anti-mitotic drugs^18^. Therefore, we hypothesized that up-regulation of MYC in KRAS^G12D^-expressing cells might result in elevated expression of pro-apoptotic proteins and/or reduced expression of anti-apoptotic proteins, leading to accelerated cell death after challenge with anti-mitotic drugs. To test this hypothesis, we transfected doxycycline-treated HeLaG12D cells with siRNA oligos targeting *MYC*. Twenty-four hours after transfection, cells were treated with paclitaxel and immediately monitored by phase-contrast time-lapse imaging. As shown in Fig. 4a, MYC knockdown rescued mitotic cell death in KRAS^G12D^-expressing HeLa cells to levels comparable to those in non-induced cells. Apoptosis induction, as measured by PARP cleavage, was also rescued in Monastrol-treated HeLaG12D cells transfected with two different siRNA oligos targeting *MYC* (Supplementary Fig. S8a; compare lane 6 with lanes 12 and 18).

The prevailing model of cell fate regulation in response to anti-mitotic drugs hints at the existence of a yet unidentified death signal which accumulates during drug-induced mitotic arrest^18^,^35^. In order to test the possibility that MYC might mediate such a death signal, and to elucidate the mechanism by which it might do so, we tested whether MYC accumulates during paclitaxel-induced mitotic arrest. We monitored MYC protein levels in HeLaG12D cells upon mitotic entry, in the presence of paclitaxel, following release from a single thymidine block. Strikingly, MYC levels dropped rapidly upon entry into mitosis in both doxycycline-treated and untreated cells: by 12 h post-release (when ∼80% cells had already entered mitosis), MYC levels were almost undetectable (Supplementary Fig. S8b,c). This result indicates that MYC protein levels do not increase during mitotic arrest. On the contrary, they decrease rapidly. These findings therefore suggest that MYC abundance during mitosis does not determine cell fate upon exposure to anti-mitotic drugs.

Several members of the BCL-2 protein family have been involved in determining the response to anti-mitotic agents, including anti-apoptotic proteins BCL-XL and MCL-1 (reviewed in^35^). Since transcription of several of these genes is controlled by MYC^31^,^32^,^33^,^34^, we reasoned that MYC-mediated regulation of death in response to anti-mitotics might be dependent on regulation of expression of one or more of these apoptotic proteins. Interestingly, we found that BCL-XL expression was lower in doxycycline-treated HeLaG12D cells transfected with control siRNA oligos (Fig. 4b), thus providing a potential explanation for the increased lethality of KRAS^G12D^-expressing HeLa cells, both in the absence and in the presence of anti-mitotic drugs (see Fig. 3 and Supplementary Fig. S7). Moreover, we found that expression of BCL-XL and MCL-1 was up-regulated following MYC knockdown (Fig. 4b). Conversely, expression of pro-apoptotic protein BIM, which has also been involved in determining paclitaxel sensitivity, was severely down-regulated following MYC knockdown, especially following doxycycline treatment (Fig. 4b). These results suggest KRAS^G12D^ controls expression of anti-apoptotic protein BCL-XL, thus generating a pro-death environment that can be exploited by anti-mitotic drug treatment. Furthermore, our results, in agreement with what has been recently reported^18^, indicate MYC controls the expression of a network of proteins with known roles in determining apoptotic response to anti-mitotic agents. Importantly, by co-transfecting HeLaG12D cells with siRNA oligos targeting *BCL-XL* and *MYC*, we found that the rescue of cell death observed following MYC knockdown was abolished by simultaneous BCL-XL depletion (Fig. 4c,d). Not only was death in mitosis enhanced, but so was the death of daughter cells upon mitotic exit (Fig. 4e). These results indicate that MYC-mediated down-regulation of anti-apoptotic protein BCL-XL mediates sensitisation to anti-mitotic drugs induced by KRAS^G12D^. Whether down-regulation of MCL-1 and/or up-regulation of BIM play a role in this context is still unclear.

### *KRAS*-mutant cancer cells are not selectively sensitive to anti-mitotic drugs

**Figure 5:**
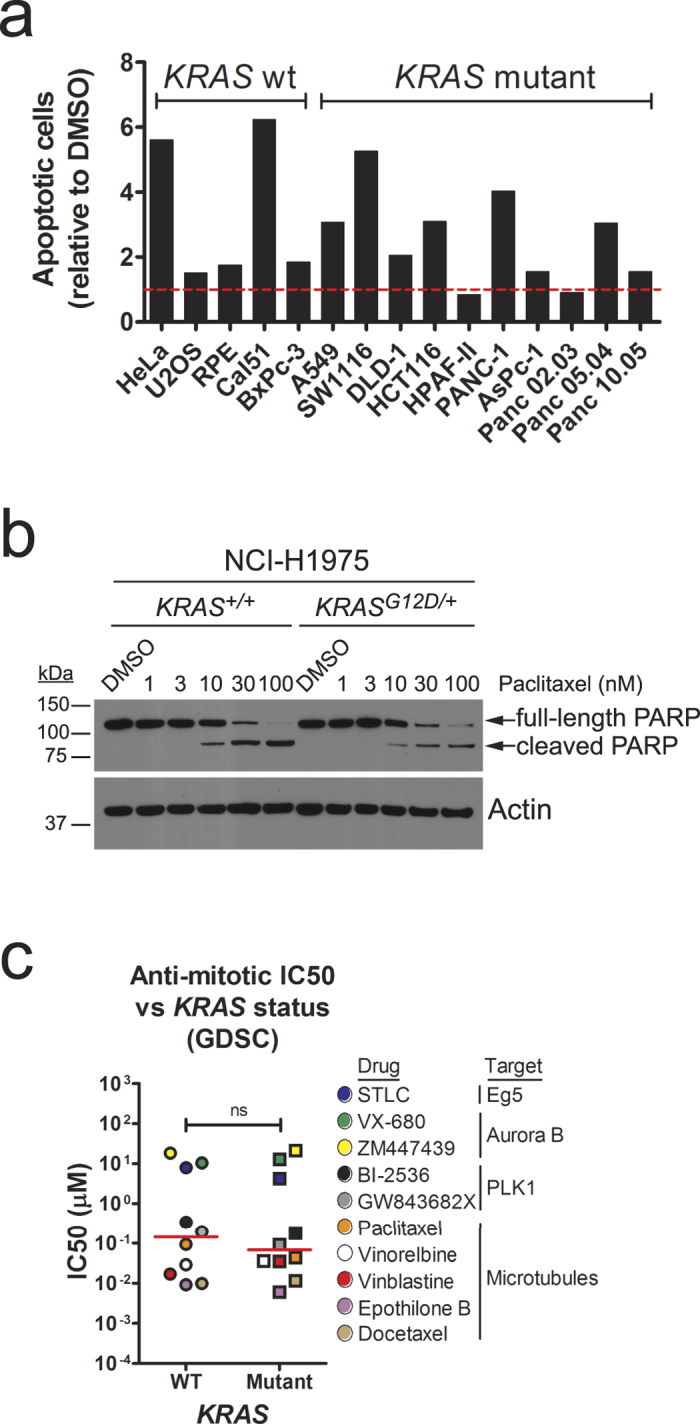
*KRAS*-mutant cancer cell lines are not selectively sensitive to anti-mitotic drugs. (**a**) Bar graph depicting induction of apoptotic cell death (measured by Annexin-V staining) of a panel of cell lines (5 wild-type, 10 mutant for *KRAS*) treated with 5 μM STLC for 48 hours. Values are represented as relative to the number of apoptotic cells in DMSO control-treated cells (shown by dashed horizontal red line). (**b**) Immunoblots of isogenic *KRAS*^+/+^ and *KRAS*^*G12D*/+^ NCI-H1975 cells treated with paclitaxel for 48 hours. Protein lysates were probed with the indicated antibodies. (**c**) Scatter dot plot depicting half-maximal inhibitory concentration (IC50) values for *KRAS* wild-type vs. mutant cancer cell lines treated with the indicated anti-mitotic drugs. Data was obtained from the Genomics of Drug Sensitivity in Cancer database (GDSC; http://www.cancerrxgene.org/). Red bars depict median values. *ns*, not significant (Wilcoxon t-test).

The MYC-dependent increased sensitivity to anti-mitotic chemotherapeutics in KRAS^G12D^-expressing HeLa cells prompted us to evaluate the possibility of using these widely drugs to selectively kill *KRAS*-mutant tumours, a strategy previously suggested by others^2^. For this purpose, we analysed a panel of *KRAS*-mutant cancer cell lines for their response to a 48-hour treatment with STLC. Apoptotic cell death was measured by Annexin-V staining, and 5 cell lines encoding wild-type KRAS were used as controls. As shown in Fig. 5a, we observed no correlation between response to STLC treatment and *KRAS* mutational status.

We reasoned that genetic alterations in these cancer cell lines other than mutations in *KRAS* might affect their response to anti-mitotics. To rule this out, and to monitor the role exclusively of KRAS^*G12D*^, we again used the isogenic pair NCI-H1975 *KRAS*^+/+^ and *KRAS*^*G12D*/+^. Strikingly, NCI-H1975 *KRAS*^*G12D*/+^ cells were slightly less sensitive to both paclitaxel and STLC when compared to their wild-type isogenic counterparts (Fig. 5b and Supplementary Fig. S9a,b). We note that this small difference might be due to differences in proliferation rate. Indeed, NCI-H1975 *KRAS*^*G12D*/+^ cells grow slightly slower than NCI-H1975 *KRAS*^+/+^ cells (Supplementary Fig. S9c). Similarly, no apparent increase in sensitivity to STLC was observed when using a different isogenic pair of cancer cell lines: SW48 *KRAS*^+/+^ and *KRAS*^*G12D*/+^ (Supplementary Fig. S9d). Interestingly, no differences in the response to STLC, measured by a short-term apoptosis assay (Supplementary Fig. S9e) or by a long-term colony forming assay (Supplementary Fig. S9f), were observed when comparing isogenic colorectal cancer cell lines HCT116 *KRAS*^+/−^ and *KRAS*^+/*G13D*^, which were previously used to uncover sensitivity to several mitotic perturbations such as inhibition of PLK1^2^. Note that, although we cannot rule out the possibility that differences in methodology may explain the apparent discrepancy between our study and that of Luo and colleagues^2^, our results indicate that mutations in *KRAS* do not universally confer sensitivity to anti-mitotic drug treatments. An alternative explanation could be that cells’ response to anti-mitotic agents might be differentially regulated by different mutations in *KRAS* (G12D in NCI-H1975 and SW48 isogenic pairs, as opposed to G13D in HCT116), consistent with the notion that different *KRAS* mutants generate distinct signalling network signatures^36^.

We extended our analysis to a larger panel of cancer cell lines, by surveying the Genomics of Drug Sensitivity in Cancer database^37^, which contains information for 665 cell lines and 141 drugs, 10 of which are anti-mitotics. We sub-classified the cell lines into those wild-type or mutant for *KRAS*, using the associated genomic data, then obtained half-maximal inhibitory concentration (IC50) for each anti-mitotic drug (obtained using 72-hour viability assays) and plotted the mean IC50 values for each drug against *KRAS* mutational status. Strikingly, we observed no correlation between sensitivity to anti-mitotic drugs and KRAS status (Fig. 5c, Supplementary Table S2 and Supplementary Fig. S10a). Similar results were obtained when analysing drug response data for the NCI-60 cell line panel (Supplementary Fig. S10b^38^).

### *KRAS*-mutant cells expressing high levels of *MYC* are sensitive to anti-mitotic drugs

**Figure 6:**
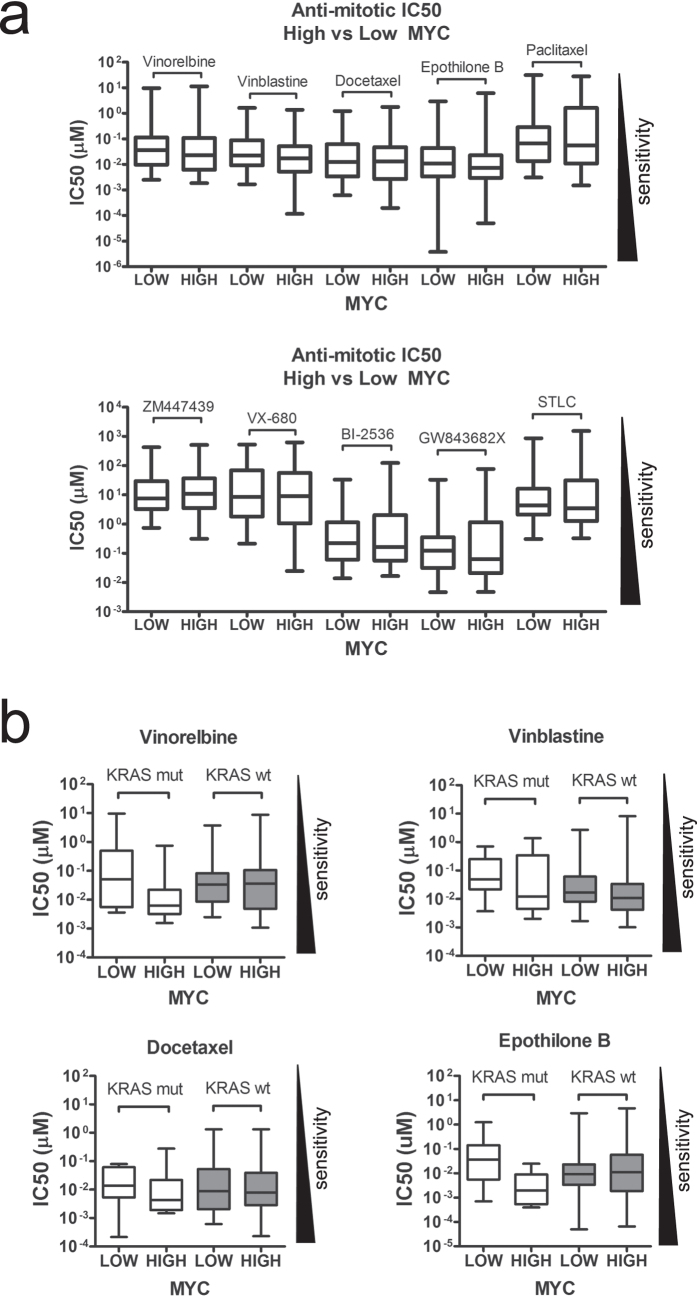
*KRAS*-mutant cancer cell lines expressing high levels of *MYC* are sensitive to anti-mitotic drugs. (**a**) Scatter dot plot depicting IC50 values for the top 50 *MYC*-expressing cancer cell lines (MYC high) vs. the bottom 50 *MYC*-expressing lines (MYC low) treated with the indicated anti-mitotic agents. Data was obtained from the Genomics of Drug Sensitivity in Cancer database. Red bars depict median values. No statistically significant differences were observed in any pair-wise comparison (Mann Whitney test). (**b**) Box-and-whiskers graphs depicting IC50 values for the indicated anti-mitotic drugs for the top 10 *MYC*-expressing *KRAS*-mutant cancer cell lines vs. the bottom 10 *MYC*-expressing lines, as well as the top 50 *MYC*-expressing *KRAS*-wild-type (wt) cancer cell lines vs. the bottom 50 *MYC*-expressing lines.

It has been recently suggested that elevated MYC levels might predict anti-mitotic drug sensitivity^18^, a hypothesis supported by our own observations (see Fig. 4). To test this hypothesis, we again utilised the Genomics of Drug Sensitivity database, where *MYC* gene expression data are available for 625 out of a total of 665 cell lines tested. Cell lines were arranged according to their *MYC* expression levels, and IC50 values for the 10 anti-mitotic drugs used in this database were plotted for the top 50 and the bottom 50 *MYC*-expressing cell lines. Unexpectedly, no significant differences in IC50 values were observed when comparing cell lines with elevated levels of *MYC* against those with low *MYC* expression (Fig. 6a), questioning the proposal that *MYC* expression alone is a reliable biomarker for anti-mitotic drug responses, and suggesting that other factors must also be involved.

We then sub-classified these 625 cell lines into wild-type or mutant for *KRAS*, and plotted IC50 values for the microtubule-interfering agents vinblastine, vinorelbine, docetaxel and epothilone-B, and the Aurora B inhibitor ZM447439, in relation to *MYC* gene expression as above. We note that IC50 values for the other 5 anti-mitotic drugs used in this database (STLC, VX-680, BI-2536, GW843682X and paclitaxel) were restricted to only ~20 *KRAS*-mutant cell lines in each case, so we did not include these drugs in the analysis. *MYC* gene expression data were only available for 77 out of 85 *KRAS*-mutant cell lines in this database, so we only plotted IC50 values for the top 10 *MYC*-expressing *KRAS*-mutant cell lines compared to the bottom 10 expressing lines. Strikingly, a lower IC50 (corresponding to increased sensitivity) was observed for the top *MYC*-expressing *KRAS*-mutant cell lines compared to the bottom *MYC*-expressing lines (Fig. 6b). We note that statistical significance is only achieved with epothilone-B (p < 0.01, Mann Whitney test), but the other 3 drugs show a similar trend. Interestingly, there was no difference in the IC50 for ZM447439 (data not shown), which unlike the microtubule-targeting drugs does not prolong mitotic arrest but instead accelerates mitotic exit^39^. We then performed the same analysis on the ∼520 *KRAS*-wild-type cell lines for which *MYC* gene expression and IC50 data were available. Strikingly, no obvious differences were observed in the IC50 values for vinorelbine, vinblastine, docetaxel or epothilone-B when comparing the top *MYC*-expressing *KRAS*-wild-type cell lines with the bottom *MYC*-expressing lines (Fig. 6b). Taken together, these results suggest that *KRAS*-mutant cancer cell lines expressing high levels of *MYC* are especially sensitive to microtubule-interfering agents, opening up new avenues for therapeutic intervention. Furthermore, they suggest *MYC* expression might not serve as a sole predictor of sensitivity to anti-mitotic drugs, but instead point to a potential cooperative effect of *KRAS* mutation and high *MYC* expression in determining anti-mitotic drug response.

## Discussion

In this study, by combining the use of isogenic cell line pairs with a targeted bioinformatics analysis of a publicly available database and the study of mitosis progression in cultured epithelial cancer cells upon acute expression of oncogenic KRAS, we make several observations with important implications for cancer biology and therapy. Unexpectedly, we find no correlation between anti-mitotic drug response and *KRAS* status in archival analysis of over 600 cancer cell lines, in experimental comparison of a panel of 5 *KRAS*-wild-type and 10 *KRAS*-mutant cancer cell lines, or when measuring induction of apoptotic cell death in three independent isogenic paired cell lines treated with anti-mitotic drugs (see Fig. 5 and Supplementary Figs S9 and S10). Thus, our results – arising from three independent methods – question the proposal that anti-mitotic drugs may preferentially kill *KRAS*-mutant cancer cells, and caution against the use of *KRAS* mutational status alone as a predictive marker for patient stratification.

However, we find that acute over-expression of KRAS^G12D^ in HeLa cells leads to aberrant progression through mitosis. This phenotype is dependent on MAPK pathway activation, as it is rescued, at least partly, by MEK inhibition (see Fig. 1). Similar results have been reported when HRAS^G12V^ was over-expressed in mouse NIH/3T3 cells^13^, rat thyroid cells^14^ or primary human fibroblasts^17^. However, to our knowledge, this is the first time such phenotype is described upon expression of KRAS^G12D^, the most prevalent *RAS* mutation in cancer, in human cells. Anaphase bridges are observed in ∼15% of human pancreatic ductal adenocarcinomas (PDAs)^10^ and in PDAs and liver metastases of *Kras* and *Trp53* double mutant mice^40^. Our results suggest KRAS^G12D^ expression may play a role in the appearance of these mitotic abnormalities, possibly by de-regulating expression of one or more mitosis-related genes prior to mitotic entry (see Supplementary Fig. S5).

Interestingly, acute expression of KRAS^G12D^ in non-transformed RPE cells does not elicit the same mitotic phenotypes as in HeLa cells, probably due to a lack of full MAPK pathway activation in the former (see Supplementary Fig. S6). This result indicates that mitotic defects are not a universal phenotype downstream of KRAS^G12D^ activation; instead, they are likely to be dependent on cell type and, more specifically, on the ability of KRAS^G12D^ to activate the MAPK pathway above a certain threshold required to elicit gene expression changes which result in de-regulation of the mitotic machinery.

Notably, we find that acute expression of KRAS^G12D^ in HeLa cells also leads to increased sensitivity to anti-mitotic drugs. Our findings indicate that this phenotype is mediated by the ability of oncogenic KRAS to up-regulate MYC, which itself regulates transcription of several apoptotic genes in other contexts^31^,^32^,^33^,^34^. This is in agreement with a recent report showing that MYC is a key mediator of anti-mitotic drug responses^18^. In particular, we show here that RNAi-mediated depletion of MYC leads to up-regulation of anti-apoptotic proteins BCL-XL and MCL-1, as well as down-regulation of pro-apoptotic protein BIM. Moreover, BCL-XL knockdown abolishes the rescue of cell death observed following MYC down-regulation, suggesting a model wherein BCL-XL works downstream of MYC as a major determinant of cell death in response to anti-mitotic agents. Interestingly, previous reports suggest a role for BCL-XL in mediating anti-mitotic drug responses^41^. Our work, together with that of Topham and colleagues^18^, confirms this finding, and establishes MYC as a key regulator of cell fate in response to anti-mitotics by working upstream of BCL-XL.

Quantitative PCR analysis of *MCL-1* following MYC RNAi showed no changes at the mRNA level (data not shown), suggesting MYC regulates MCL-1 at a post-transcriptional level. Interestingly, proteasomal degradation of MCL-1, mediated by ubiquitin ligases APC/C and FBW7, has been shown to play a key role in cell fate during drug-induced mitotic arrest^42^,^43^. Whether MYC-mediated modulation of MCL-1 levels also plays a role in determining anti-mitotic drug response is yet unclear. We also find that levels of pro-apoptotic protein BIM are regulated by MYC in KRAS^G12D^-expressing HeLa cells (see Fig. 4b). Whether MYC-mediated regulation of BIM occurs at the transcriptional or post-transcriptional level, and whether this regulation plays a role in determining anti-mitotic drug response, is still unclear.

We find - as expected from the short half-life of both *MYC* mRNA and protein^44^,^45^, coupled to the near-complete cessation of *de novo* protein synthesis during mitosis^46^,^47^ - that *MYC* protein levels quickly drop during drug-induced mitotic arrest (see Supplementary Fig. S8b,c). This suggests that MYC regulates the transcription of apoptotic genes in interphase so that, upon mitotic entry, cells are primed for death if mitosis should be delayed. In other words, MYC may determine the ‘death threshold’ before cells enter mitosis. We propose that this mechanism acts in concert with other layers of regulation of death in response to anti-mitotics that directly rely on accumulation of death signals during prolonged mitotic arrest, such as SCF^FBW7^-dependent proteasomal degradation of MCL-1^42^,^43^, phosphorylation of BCL-2, BCL-XL and BID^48^,^49^, cyclin G1 over-expression^50^, caspase-9 dephosphorylation^51^ or telomere deprotection^52^. However, it is yet unclear whether these mitotic death signals co-exist in every cell, and so it is conceivable that drug responses might be regulated by different pathways in different tissues or cell types, consistent with the reported variability in anti-mitotic drug responses across multiple cell lines^53^,^54^.

Finally, we demonstrate here for the first time that *KRAS*-mutant cancer cell lines expressing elevated levels of *MYC* show increased sensitivity to several microtubule-interfering agents, opening up new avenues for therapeutic intervention and suggesting new strategies for patient stratification. Our study therefore bridges two key areas of cancer therapy: the use of anti-mitotic chemotherapeutics for the treatment of several types of cancer (where a clear understanding of the factors determining patients’ response is still lacking) and the long-standing, yet largely unsuccessful efforts to treat tumours harbouring mutant *KRAS*, which account for 20–25% of all human cancers.

## Methods

### Cell culture and reagents

Isogenic pairs NCI-H1975 *KRAS*^+/+^ and *KRAS*^*G12D*/+^, SW48 *KRAS*^+/+^ and *KRAS*^*G12D*/+^, and HCT116 *KRAS*^+/−^ and *KRAS*^+*/G13D*^ were purchased from Horizon Discovery (Cambridge, UK). All cell lines were grown in DMEM GlutaMAX supplemented with 10% foetal calf serum, 100 U/ml penicillin and 100 mg/ml streptomycin (Life Technologies), except RPE, which were cultured in DMEM:F-12 media supplemented with 0.25% sodium bicarbonate (Life Technologies), SW48 (RPMI-1640 media, Life Technologies), and Panc-02-03, Panc-05-04 and Panc-10-05 (RPMI-1640 supplemented with 20 units/ml human recombinant insulin, Sigma). *KRAS* status for all cell lines used was obtained from the Catalogue of Somatic Mutations in Cancer (COSMIC) database, version 76 (http://cancer.sanger.ac.uk/cosmic). Parental FRT/TO HeLa and RPE cell lines (kind gifts from Stephen Taylor [University of Manchester] and Jon Pines [Gurdon Institute, Cambridge], respectively) were used to generate doxycycline-inducible cell lines as described previously^55^,^56^. Stable integrants were selected with 4 μg/ml blasticidin (Invivogen) and 200 μg/ml hygromycin (Roche) for HeLa, or 10 μg/ml blasticidin and 400 μg/ml G418 (Life Technologies) for RPE. Transgene expression was achieved by treatment with 0.1 μg/ml doxycycline (Sigma). Other chemicals were obtained from the following suppliers: Tocris Bioscience (monastrol and STLC), Sigma (thymidine and paclitaxel), Cell Signalling (EGF), Calbiochem (U0126) and Selleckchem (AZD6244). Knockdown experiments were performed by transfecting cells with siRNA oligos at 25 nM (Supplementary Table S3), using jetPRIME transfection reagent (Polyplus).

### Immunoblotting

Cells were lysed in RIPA buffer and proteins quantified using BCA assay (Thermo Scientific). Proteins were loaded onto NuPAGE pre-cast gels (Life Technologies) and transferred onto HyBond ECL nitrocellulose membranes (GE Healthcare). Membranes were blocked in TBST (10 mM Tris-HCl pH 7.4, 150 mM NaCl, 0.1% Tween-20) plus 5% non-fat dry milk for 1 hour, before incubation with the antibodies listed in Supplementary Table S4, overnight at 4 °C. Following washes with TBST, membranes were incubated with HRP-conjugated secondary antibodies (GE Healthcare) and developed using SuperSignal West Pico chemiluminescent substrate (Thermo Scientific).

### Live cell imaging

Cells were grown on multi-well 1.0-mm-thick borosilicate chamber slides (Lab-Tek). Phase-contrast time-lapse microscopy was performed on a Zeiss Axiovert 200M microscope, acquiring images every 5 minutes under a 20X objective. To visualise chromatin, GFP-tagged histone H2B was subcloned from pH2B-GFP (Addgene 11680) into pcDNA3.1/puro (kind gift from Chris Sullivan, University of Texas). The H2B-GFP transgene was then stably integrated into the required cell lines, which were selected with puromycin (Invivogen). Fluorescence live-cell imaging was performed on a Leica DMI6000 microscope using a HCX PL APO objective (40X magnification, N.A. 0.85), and images acquired every 5 minutes on an Evolve 512 EMCCD camera (Photometrics) using LAS AF software, then processed and analysed with ImageJ (NIH, Bethesda, Maryland).

### Colony forming assays

Cells were treated with doxycycline for 48 hours before plating in triplicate on 6-well dishes, at a dilution of 200 cells per well. Anti-mitotic drugs were then added for 72 hours, then washed out and cells allowed to grow for a further 5 days in the presence or absence of doxycycline. Cells were then fixed in 4% formaldehyde, washed in PBS and stained with 0.1% crystal violet (Sigma) for 20 minutes before washing with water. Plates were allowed to dry overnight and colonies were counted using ColCount (Oxford Optronix).

### Flow cytometry

For measurement of apoptosis, cells were plated on 6-well dishes at a dilution of 200,000 cells per well. Anti-mitotic drugs were added the following day and cells harvested 48 hours later. Apoptotic cells were detected by Annexin-V staining using Annexin-V/FITC Apoptosis Detection Kit II (BD Biosciences), according to manufacturer’s instructions, and analysed on a Becton Dickinson LSR II flow cytometer. Data was processed with FlowJo software. For cell cycle analysis, cells were fixed in ethanol, blocked in PBS with 0.1% Triton X-100 (PBST) and 1% BSA for 30 minutes, then incubated with mouse anti-phospho-MPM2 antibody 1:500 (Millipore) for 2 h at room temperature. Following washes with blocking solution, cells were incubated with Alexa Fluor 488 secondary antibody (Life Technologies) for 1 h, washed with PBST and treated with 40 μg/ml propidium iodide (Sigma) and 200 μg/ml RNaseA (Sigma), then analysed as above.

### Immunofluorescence and image analysis

For mitotic spindle analysis, cells grown on coverslips were treated with 100 μM Monastrol for 4 h, then washed 3 times with normal growth media and released into media containing 20 μM MG132 (Calbiochem) for 2 h to block metaphase-to-anaphase transition. Cells were then permeabilised for 90 s in K-buffer (100 mM PIPES pH 6.8, 1 mM MgCl_2_, 0.1 mM CaCl_2_, 0.1% Triton X-100) to preserve K-fibres, and fixed for 10 min in 4% formaldehyde diluted in K-buffer. Following washes in PBS with 0.1% Triton X-100 (PBST), cells were blocked in PBST plus 5% milk, incubated with mouse monoclonal anti-β-Tubulin 1:1000 (clone D66, Sigma) for 30 min, then incubated with Alexa Fluor 568 secondary antibody 1:500 (Life Technologies) for another 30 min. Following washes in PBST, coverslips were mounted and counterstained with DAPI-containing VectaShield (Vector Labs). Images were acquired as 1 μm-step Z-stacks on a Leica SP5 laser scanning confocal microscope using a 100x objective (N.A. 1.4), and analysed and processed using ImageJ.

For co-staining of ERK1/2 and myc-tagged KRAS, cells grown on coverslips were fixed in 4% formaldehyde diluted in PBS for 10 min at room temperature. Following washes in PBST, cells were blocked in PBST plus 1% BSA for 30 min, then incubated with mouse monoclonal anti-Myc tag 1:500 (clone 4A6, Millipore) and rabbit monoclonal anti-ERK1/2 1:100 (clone 137F5, Cell Signalling) for 1 h at room temperature. Following washes in PBST, cells were incubated with Alexa Fluor 488 or 568 secondary antibodies diluted 1:500, then mounted as above. Images were acquired as 1 μm-step Z-stack tile scans on a Leica SP5 laser scanning confocal microscope using a 63x objective (N.A. 1.4). Nucleo-cytoplasmic ratio of ERK1/2 was measured using ImageJ. Briefly, a nuclear mask was created using the DAPI image, then superimposed on the ERK1/2 image to quantify nuclear signal. The cytoplasmic signal intensity was then quantified from the inverse image once the extracellular background signal was removed.

### Extraction of RNA and quantitative PCR

Total RNA was obtained using the RNeasy mini kit (Qiagen), according to manufacturer’s instructions, and cDNA was generated from 1 μg RNA using the Cloned AMV First-Strand cDNA Synthesis Kit (Invitrogen), using Oligo(dT)20 primers and Random Hexamers. Quantitative PCR was performed on the LightCycler 480 (Roche) using LightCycler 480 SYBR Green I Master Mix (Roche), according to the manufacturer’s recommendations. Gene-specific primers were designed manually or using Primer-BLAST (NCBI), purchased from Sigma, tested in a standard PCR reaction for generation of a single band only, and used at a final concentration of 0.25 μM (see Supplementary Table S5). Each sample was run in triplicates in a 96-well LightCycler 480 Multiwell Plate (Roche), and mRNA levels were estimated by normalising Cp values for each gene compared to Cp values of the housekeeping gene TATA-box binding protein (TBP), using the equation 2^[Cp(ref)-Cp(target)]^, where *ref* is the reference gene (TBP) and *target* is each particular target gene.

### Statistical analyses

All statistical analyses were performed using GraphPad Prism version 5 (GraphPad Software).

## Additional Information

**How to cite this article**: Perera, D. and Venkitaraman, A. R. Oncogenic KRAS triggers MAPK-dependent errors in mitosis and MYC-dependent sensitivity to anti-mitotic agents. *Sci. Rep.* **6**, 29741; doi: 10.1038/srep29741 (2016).

## Supplementary Material

Supplementary Information
