# Supplementary material for: Oncogenic KRAS triggers MAPK-dependent errors in mitosis and MYC-dependent sensitivity to anti-mitotic agents
Source: Sci Rep. 2016 Jul 14;6:29741. doi: 10.1038/srep29741 (PMC4944194; doi:10.1038/srep29741)
Supplement: Supplementary Information [file srep29741-s1.pdf]

SUPPLEMENTARY INFORMATION

Oncogenic KRAS triggers MAPK-dependent errors in mitosis and MYC-dependent sensitivity to anti-mitotic agents

David Perera and Ashok R. Venkitaraman*

Contents:

10 Supplementary Figures

5 Supplementary Tables

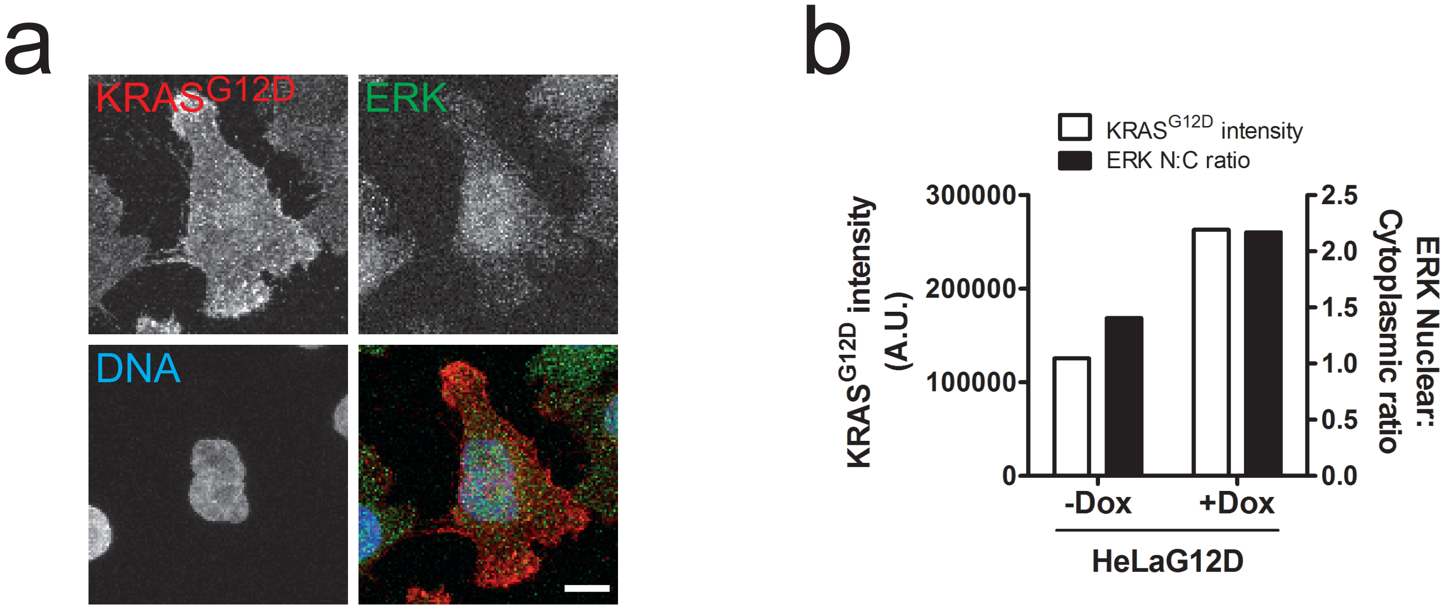

Supplementary Figure S1. KRAS^{G12D} expression leads to nuclear translocation of ERK1/2 in HeLa cells. (a) Maximum projections of confocal images of a HeLaG12D cell treated with doxycycline for 24 h, showing staining for myc-tagged KRAS^{G12D} (red; note that signal appears to concentrate in areas of the plasma membrane), endogenous ERK1/2 (green; mainly localised in the nucleus) and DNA (blue). Scale bar, 10 μm. **(b)** Bar graph depicting KRAS^{G12D} pixel intensity (left Y axis) and nucleo-cytoplasmic (N:C) ratio of ERK1/2 (right Y axis) from tile scan images of HeLaG12D cells treated and stained as in (a). 322-353 cells were analysed for each condition.

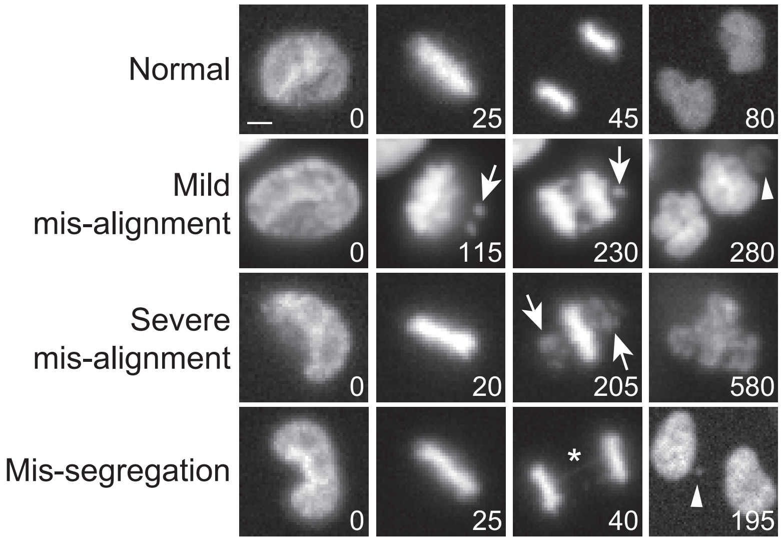

Supplementary Figure S2. Representative images of GFP-H2B-expressing HeLaG12D cells, from time-lapse movies analysed in Fig. 1c,d. Arrows depict unaligned chromosomes; asterisk shows an anaphase bridge; arrowheads mark micronuclei appearing after division. Numbers denote time (in minutes) from NEB. Scale bar, 5 μm.

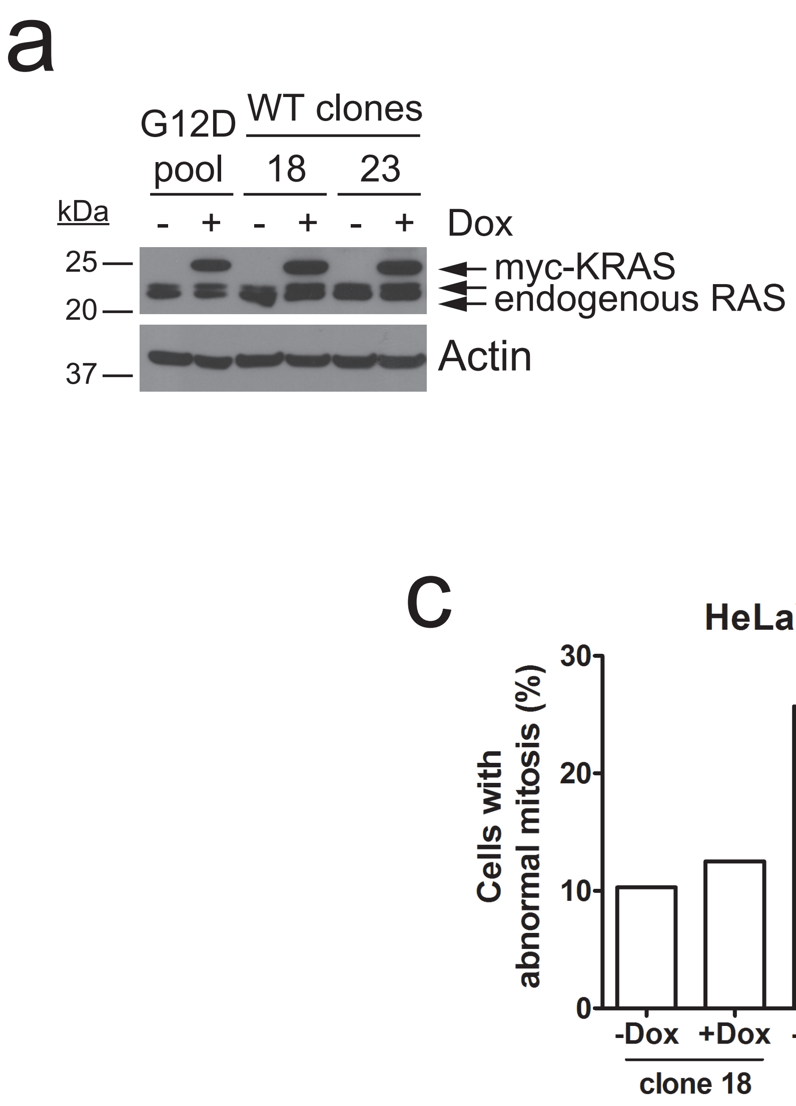

Supplementary Figure S3. KRAS^{WT} expression does not elicit mitotic defects in HeLa cells. (a) Immunoblot analysis of HeLaG12D cells and two independent HeLaWT clones treated with doxycycline for 24 hours and probed for RAS and Actin. **(b)** The same two HeLaWT clones expressing GFP-H2B were treated with doxycycline for 24 hours then monitored by time-lapse microscopy for a further 24 hours. The scatter dot plot shows time spent in mitosis (scored as the time taken from NEB to anaphase onset), represented in minutes. 34-40 cells were analysed for each condition. Horizontal bars represent mean values. **(c)** Bar graph depicting the percentage of cells with abnormal division from the time-lapse movies in (b).

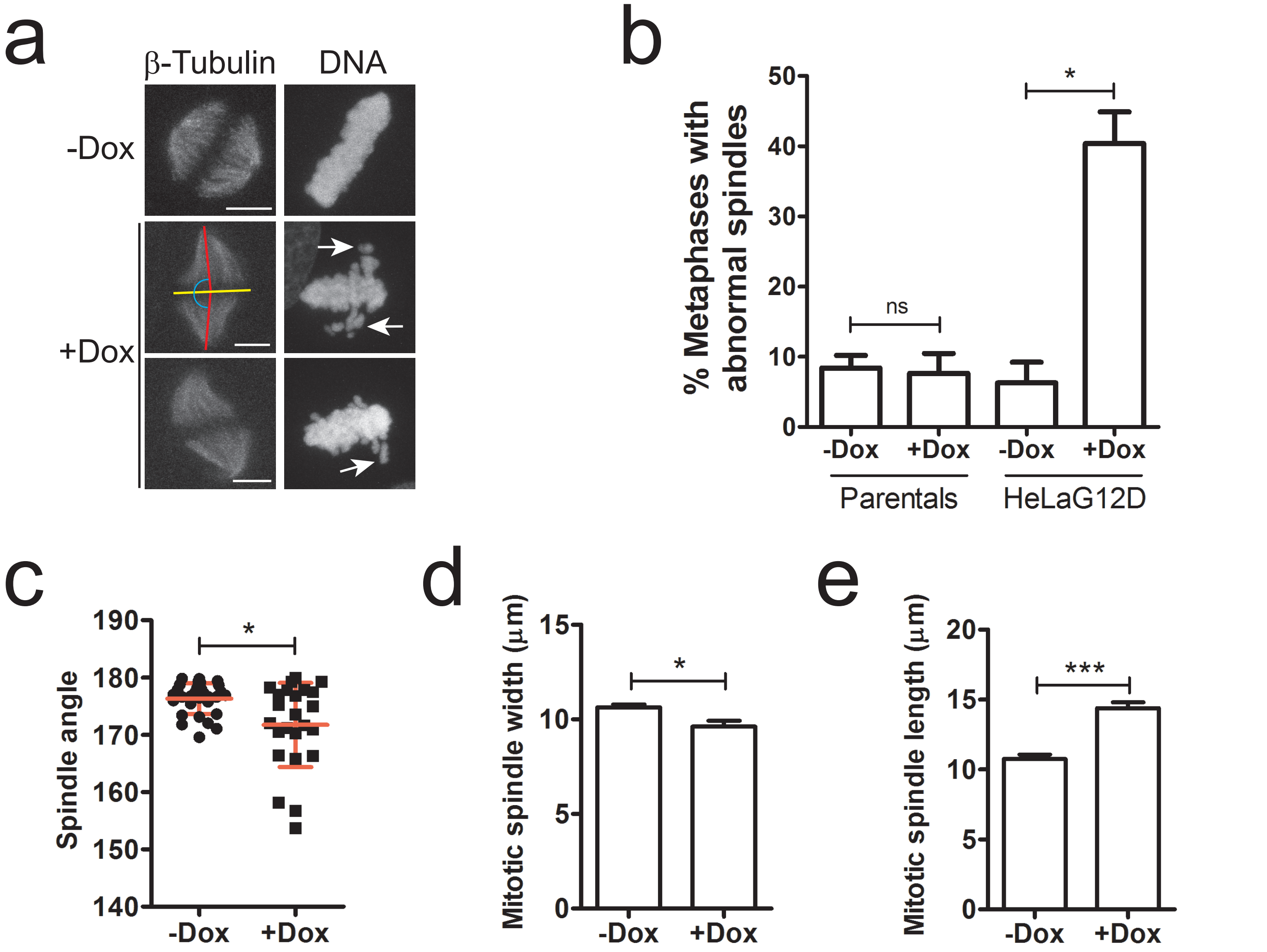

Supplementary Figure S4. KRAS^{G12D} expression leads to defective mitotic spindle morphology in HeLa cells. (a) Maximum projections of representative confocal images of HeLaG12D cells, left untreated or treated with doxycycline for 48 h, following Monastrol wash-out into MG132, showing mitotic spindles (stained with β-Tubulin) and DNA (stained with DAPI). Arrows depict unaligned chromosomes. The yellow line depicts the position of the metaphase plate (where the majority of chromosomes are positioned). Red lines connect the centre of the metaphase plate to each spindle pole. Blue arc represents the angle measured to score orientation of the spindle with respect to the poles. Scale bar, 5 μm. **(b)** Bar graph depicting the percentage of metaphases with abnormal (i.e. mal-orientated) mitotic spindles. Bars represent mean values ± S.E.M. (n=3 independent experiments). *ns*, not significant; *, $p<0.05$ (paired t-test). **(c)** Scatter dot plot showing mitotic spindle angle of individual HeLaG12D cells treated and imaged as in (a). Red lines depict mean values ± S.D. 24-30 cells were analysed per condition. *, $p<0.05$ (Mann Whitney test). **(d,e)** Bar graphs representing mean values ± S.E.M. of mitotic spindle width (d) and length (e), obtained from confocal images as in (a). 18 cells were analysed per condition. *, $p<0.05$; ***, $p<0.0001$ (Mann Whitney test).

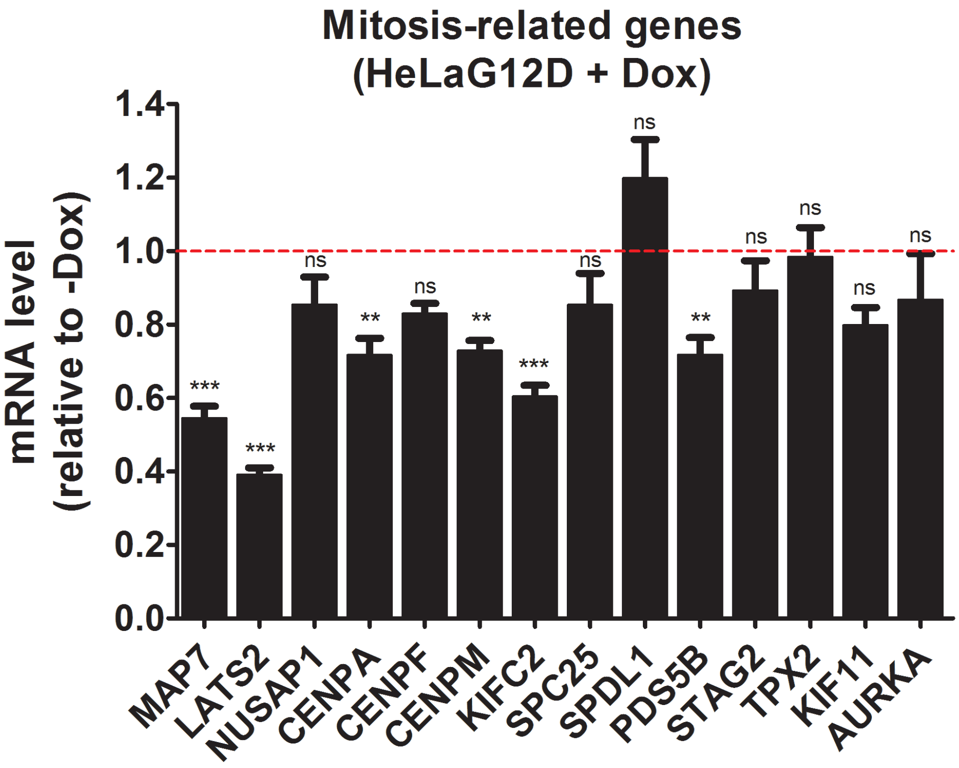

Supplementary Figure S5. KRAS^{G12D} expression leads to de-regulated expression of several genes with known roles in mitosis. Bar graph depicting mRNA levels of 14 mitosis-related genes in doxycycline-treated HeLaG12D cells 6 h following release from a double thymidine block. >80% cells were in G2 in these conditions (data not shown). Bars represent mean values ± S.E.M. (n=3 independent experiments), all relative to untreated controls (-Dox). *ns*, not significant; **, $p<0.01$; ***, $p<0.001$ (Two-way ANOVA).

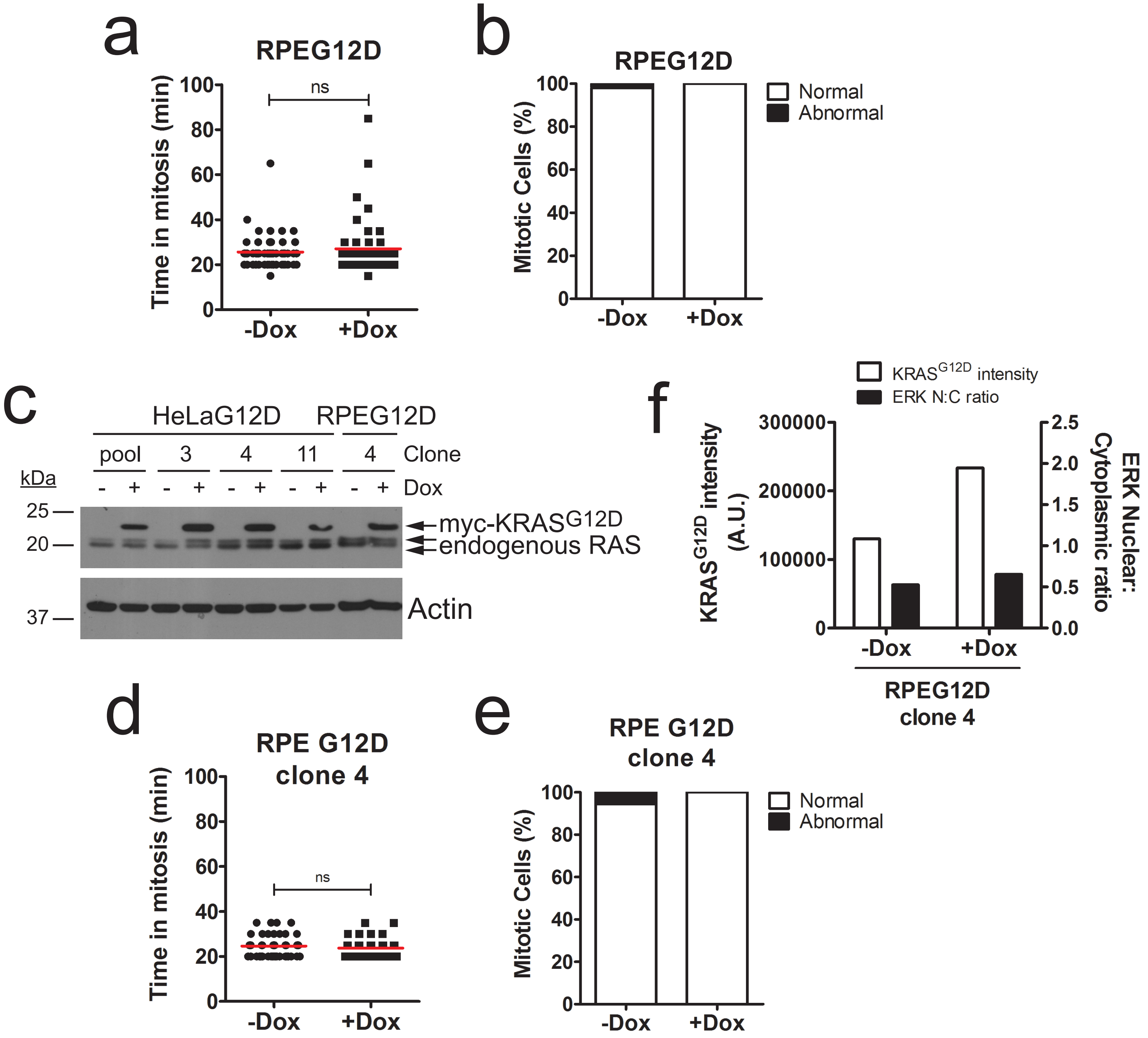

Supplementary Figure S6. KRAS^{G12D} expression does not elicit mitotic defects in RPE cells. (a) RPEG12D cells expressing GFP-H2B were treated with doxycycline for 24 hours then monitored by time-lapse microscopy for a further 24 hours. The scatter dot plot shows time spent in mitosis (scored as the time taken from NEB to anaphase onset), represented in minutes. 50 cells were analysed for each condition. Red lines represent mean values. *ns*, not significant (Mann Whitney test). **(b)** Bar graph depicting the percentage of cells with normal or abnormal division from the time-lapse movies in (a). **(c)** Immunoblot analysis of HeLaG12D cells (pool and 3 independent clones), as well as RPEG12D clone 4, treated with doxycycline for 24 hours and probed for RAS and Actin. **(d)** GFP-H2B-expressing RPEG12D clone 4 was treated with doxycycline for 24 hours then monitored by time-lapse microscopy for a further 24 hours. The scatter dot plot shows time spent in mitosis, represented in minutes. Data was obtained from 2 independent experiments, and >50 cells were analysed for each condition. Red lines represent mean values. *ns*, not significant (Mann Whitney test). **(e)** Bar graph depicting the percentage of cells with normal or abnormal division from the time-lapse movies in (d). **(f)** Bar graph depicting KRAS^{G12D} pixel intensity (left Y axis) and nucleo-cytoplasmic (N:C) ratio of ERK1/2 (right Y axis) from tile scan images of RPEG12D clone 4 cells treated with doxycycline for 24 h, then stained and analysed as in Supplementary Fig. S1a. 354-427 cells were analysed for each condition.

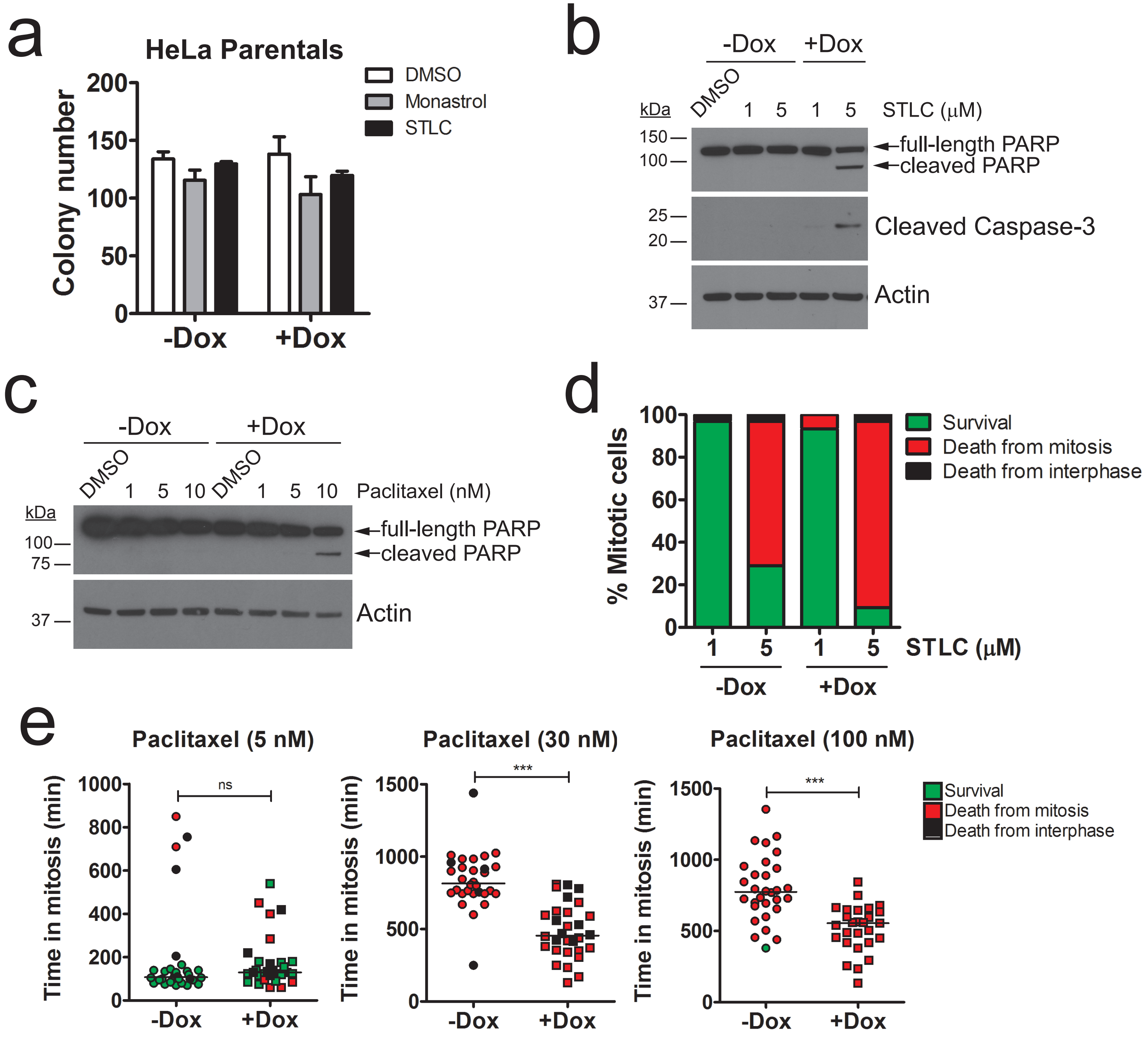

Supplementary Figure S7. Characterisation of anti-mitotic drug response in HeLaG12D and parental cells. (a) Bar graph depicting the number of colonies in HeLa FRT/TO parental cells pre-treated with doxycycline for 48 hours, then treated with 100 μM Monastrol or 5 μM STLC for 3 days and cultured in the absence of drugs for a further 5 days. Bars represent mean values ± S.E.M. (n=3 independent experiments). **(b)** Immunoblot of HeLaG12D cells treated with doxycycline for 48 hours, then with STLC for a further 24 hours. Protein lysates were probed for PARP and cleaved (i.e. active) Caspase-3. Actin was used as a loading control. **(c)** Immunoblot of HeLaG12D cells treated with doxycycline for 48 hours, then with paclitaxel for a further 28 hours. Protein lysates were probed with the indicated antibodies. **(d)** Stacked bar graph representing the fate of HeLaG12D cells pre-treated with doxycycline, then challenged with two different concentrations of STLC and monitored by phase-contrast time-lapse microscopy. Bar colors represent cell fates as described in Fig. 3c. **(e)** HeLaG12D cells were pre-treated with doxycycline then incubated with 5 (left panel), 30 (middle panel) or 100 (right panel) nM paclitaxel and filmed by phase-contrast time-lapse microscopy. Scatter dot plot shows time from mitotic entry (NEB) to either mitotic exit or death (whatever comes first). Dot colors reflect the fate of individual cells as in Fig. 3c. Only cells entering mitosis were analysed (n=27-30 per condition). Horizontal bars represent median values. *ns*, not significant; ***, p<0.0001 (Mann Whitney test).

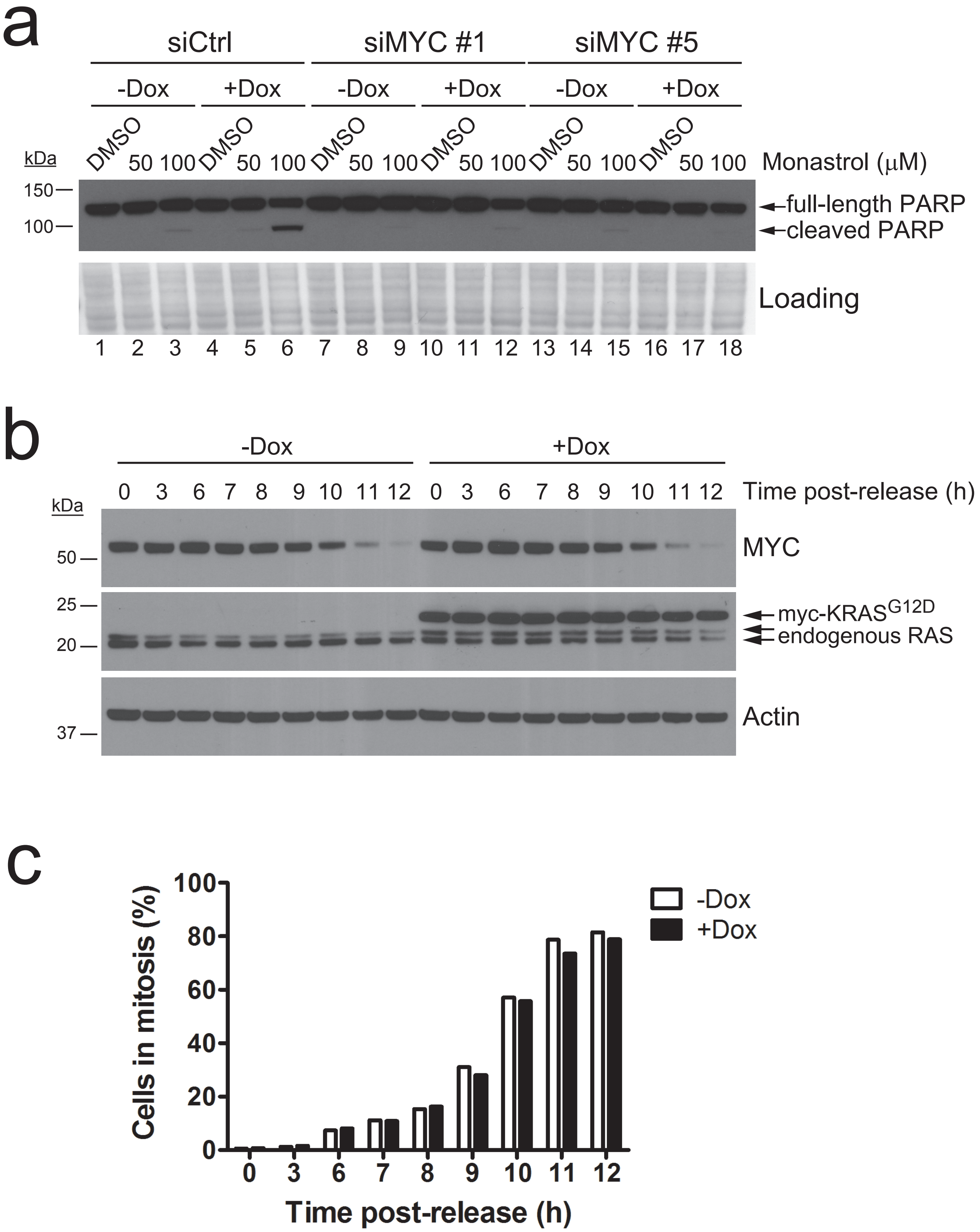

Supplementary Figure S8. MYC regulates cell death in response to Monastrol and its levels decrease rapidly upon mitotic entry. (a) Immunoblot analysis of HeLaG12D cells pre-treated with doxycycline for 48 hours, transfected with control or MYC siRNA oligos, then challenged with Monastrol for 24 hours and probed for PARP. Equal amounts of protein lysates were loaded for each sample and Ponceau staining was used as a loading control, as Actin levels decreased in samples with significant levels of death. **(b)** HeLaG12D cells were pre-treated with doxycycline before blocking in early S-phase by addition of thymidine. Sixteen hours later, cells were released into S-phase in the presence of 100 nM paclitaxel and collected at the indicated times. Protein lysates were then probed with the indicated antibodies. **(c)** Bar graph depicting the percentage of cells in mitosis, as determined by phospho-MPM2 staining, from the samples in (b).

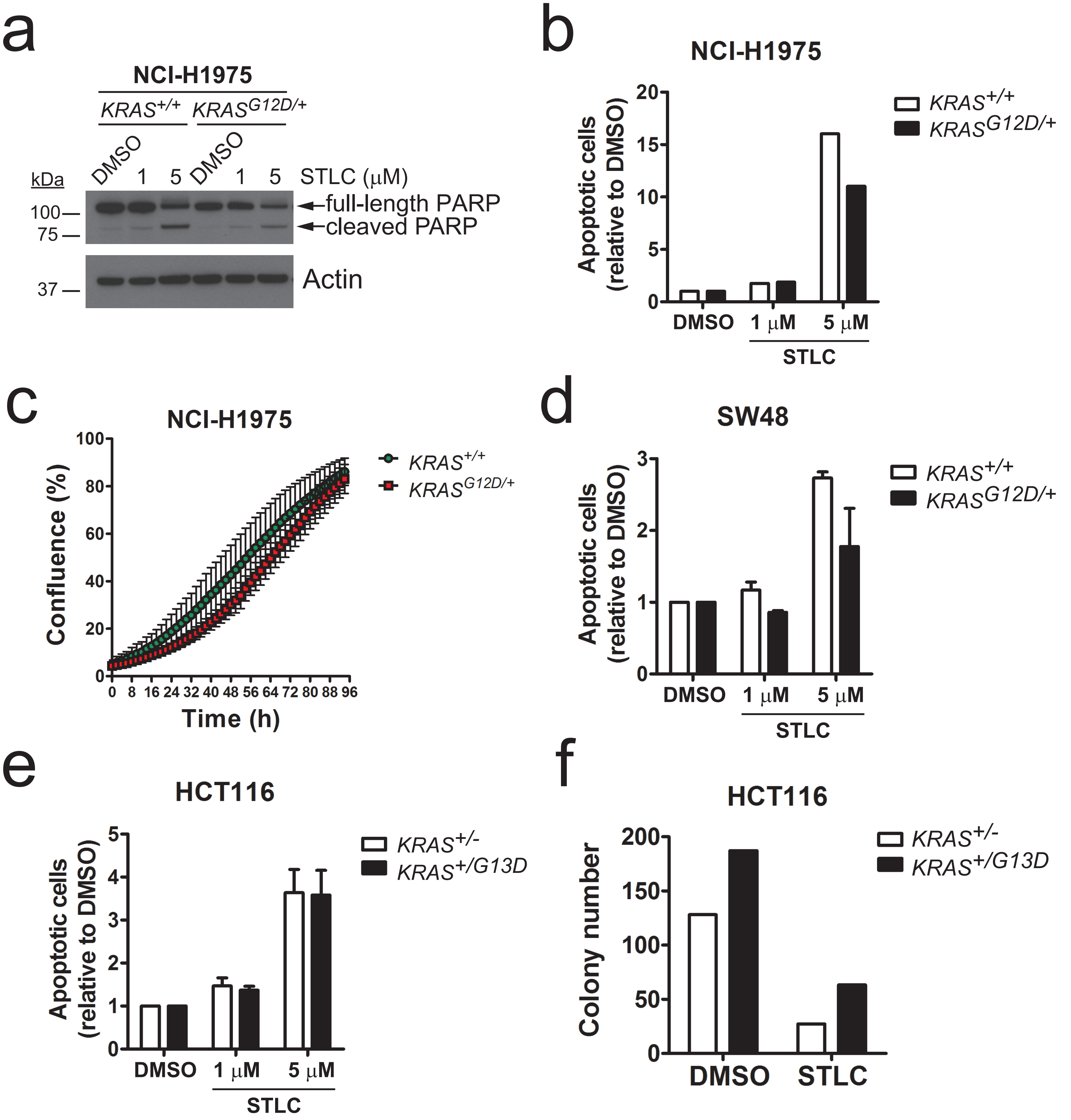

Supplementary Figure S9. Analysis of anti-mitotic drug response in isogenic paired cell lines. (a) Immunoblots of isogenic NCI-H1975 *KRAS*$^{+/+}$ and *KRAS*$^{G12D/+}$ cells treated with STLC for 48 hours, then probed with the indicated antibodies. **(b)** Bar graph depicting induction of apoptotic cell death (measured by Annexin-V staining) of isogenic NCI-H1975 *KRAS*$^{+/+}$ and *KRAS*$^{G12D/+}$ cells treated with DMSO, 1 µM or 5 µM STLC for 48 hours (normalised to DMSO control). **(c)** Growth curves of NCI-H1975 *KRAS*$^{+/+}$ and *KRAS*$^{G12D/+}$ cells. Cells were plated at 5000 cells per well in 24-well dishes and growth was monitored in an IncuCyte^{TM} (Essen BioScience) for up to 4 days. Shown are mean values ± S.E.M. from 3 independent experiments. **(d)** Bar graph depicting induction of apoptotic cell death of isogenic SW48 *KRAS*$^{+/+}$ and *KRAS*$^{G12D/+}$ cells treated as in (b). Bars represent mean values ± S.D. (n=2 independent experiments). **(e)** Bar graph depicting induction of apoptotic cell death of isogenic HCT116 *KRAS*$^{+/-}$ and *KRAS*$^{+/G13D}$ cells treated as in (b). Bars represent mean values ± S.D. (n=2 independent experiments). **(f)** Bar graph depicting the number of colonies in isogenic HCT116 *KRAS*$^{+/-}$ and *KRAS*$^{+/G13D}$ cells treated with DMSO or 1 µM STLC for 3 days and cultured in the absence of drugs for a further 5 days. Note that higher concentrations of STLC resulted in no colonies forming for either cell line.

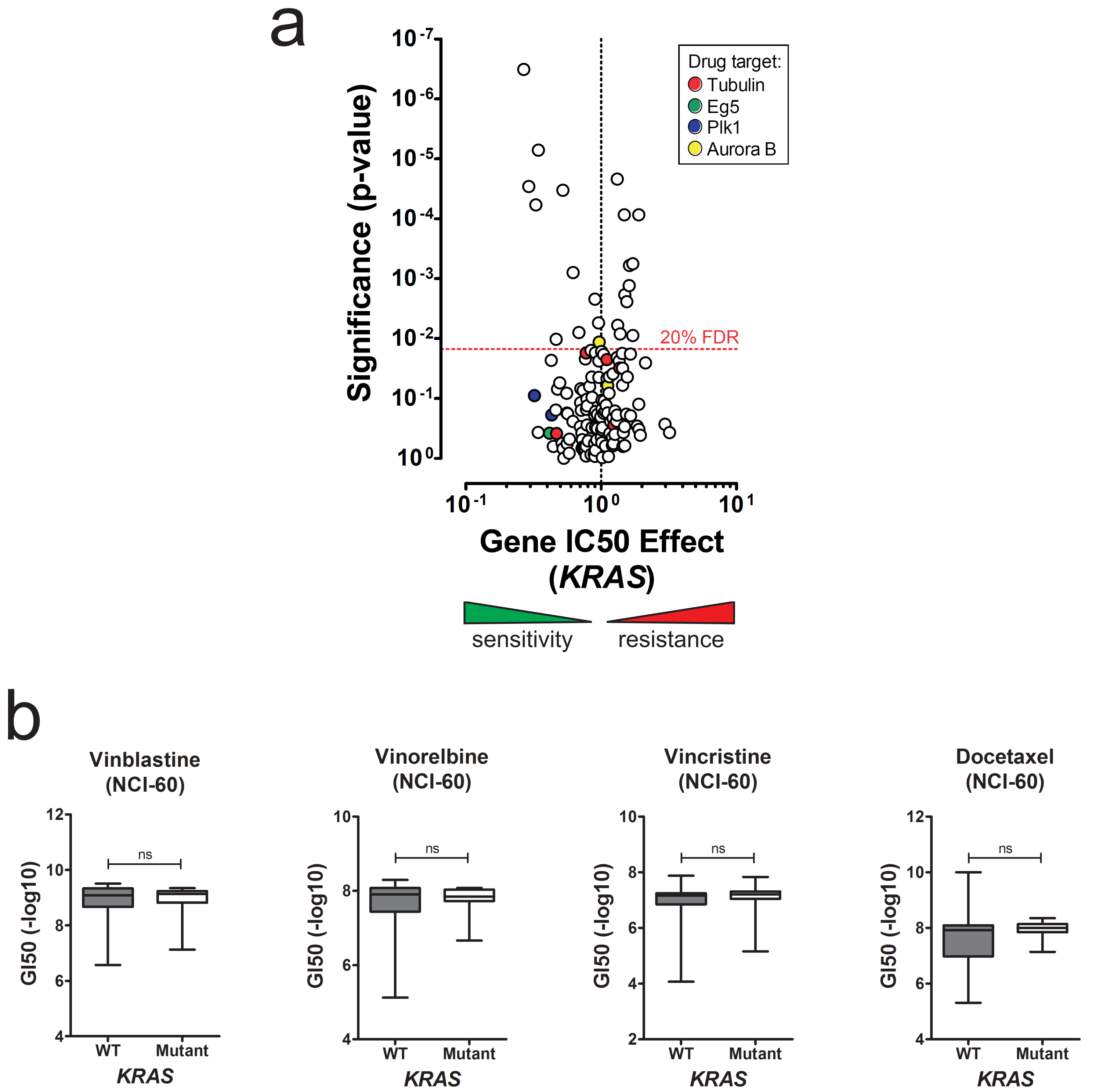

Supplementary Figure S10. Additional analysis of drug response databases. (a) Volcano plot showing the gene IC50 effect and significance of *KRAS*-drug associations. Each circle represents a single drug effect. The x axis represents the magnitude of the effect that genetic events have on cell line IC50 values in response to a drug. IC50 values were correlated with the status of *KRAS* using a two-way multivariate ANOVA, with mutation status and tissue type as factors. Numbers less than 1 indicate drug sensitivity; numbers greater than 1 indicate drug resistance. The y axis represents the p-value from the two-way multivariate ANOVA of a drug-gene interaction on an inverted log 10 scale. The dashed red line represents a Benjamini-Hochberg multiple testing correction threshold with a false discovery rate (FDR) of 20%. Note that all gene-drug associations for anti-mitotic drugs (coloured in red, green, blue or yellow, depending on their intracellular target) fall below the significance threshold, with the exception of ZM447438 (Aurora B inhibitor), which is just above the threshold. Data was obtained from the Genomics of Drug Sensitivity in Cancer database (http://www.cancerrxgene.org). **(b)** Box-and-whisker graphs depicting negative log10 values of half-maximal growth inhibitory concentrations (GI50) for *KRAS* wild-type vs. mutant cancer cell lines treated with the indicated drugs. Data was obtained from the CellMiner database (http://discover.nci.nih.gov/cellminer/home.do). *ns*, not significant (Mann Whitney test).

	Fate of daughter cells (P1)				*Fate of daughter cells (P2)*		
**Type of mitosis**	**Daughter cells (P1)**	**Survive**	**Divide again**	**Die**	**Daughter cells (P2)**	**Survive**	**Die**
Normal	63	0	89% (56/63)	11.1% (7/63)	87	92% (80/87)	8% (7/87)
Abnormal	51	6	74.5% (38/51)	13.7% (7/51)	46	84.8% (39/46)	15.2% (7/46)

Supplementary Table S1. Fate of doxycycline-treated HeLaG12D cells following release from a double thymidine block and filmed for 48 hours. 61 dividing cells were analysed, of which 33 divided normally (Normal) and 28 displayed signs of chromosome mis-alignment and/or mis-segregation (Abnormal). Note that some daughter cells disappeared from the field of view and therefore were not included in the analysis.

**Drug**	**Target**	**IC50 *KRAS* wild-type**	**IC50 *KRAS* mutant**	**Total cell lines analysed**	**No. *KRAS* mutant cell lines**
S-Trityl-L-cysteine	Eg5	7.885	4.177	355	26
VX-680	Aurora B	10.384	12.869	355	26
ZM-447439	Aurora B	18.232	21.222	642	90
BI-2536	PLK1	0.338	0.179	356	26
GW843682X	PLK1	0.194	0.092	356	26
Paclitaxel	Microtubules	0.093	0.043	356	26
Vinorelbine	Microtubules	0.029	0.035	672	96
Vinblastine	Microtubules	0.016	0.034	663	95
Epothilone B	Microtubules	0.009	0.005	665	95
Docetaxel	Microtubules	0.009	0.011	663	95

Supplementary Table S2 (Related to Fig. 5c). Half-maximal inhibitory concentration (IC50) values (in μM) for *KRAS* wild-type vs. mutant cancer cell lines treated with the indicated anti-mitotic drugs. Data was obtained from the Genomics of Drug Sensitivity in Cancer database (http://www.cancerrxgene.org/).

Oligo name	Target gene (protein)	Target sequence (5' – 3')	Supplier
AllStars	None (negative control)	n.a.	QIAGEN
Hs_MYC_1	*MYC*	CCCAAGGTAGTTATCCTTAAA	QIAGEN
Hs_MYC_5	*MYC*	GATCCCGGAGTTGGAAAACAA	QIAGEN
Hs_BCL2L1_2	*BCL2L1* (BCL-XL)	CTGCTTGGGATAAAGATGCAA	QIAGEN

Supplementary Table S3. List of siRNA oligos used in this study. *n.a.*, not available.

Antibody name	Catalogue number	Supplier
Rabbit polyclonal anti-MYC	06-340	Millipore
Mouse monoclonal anti-RAS	1862335	Thermo Scientific (Life Technologies)
Mouse monoclonal anti-Phospho-p44/42 MAPK (ERK1/2) (Thr202/Tyr204) (clone E10)	9106	Cell Signalling
Rabbit monoclonal anti-p44/42 MAPK (ERK1/2) (clone 137F5)	4695	Cell Signalling
Mouse monoclonal anti-PARP (clone C2-10)	556362	BD Biosciences
Rabbit polyclonal anti-active Caspase-3	ab13847	Abcam
Rabbit monoclonal anti-BCL-XL (clone 54H6)	2764	Cell Signalling
Rabbit polyclonal anti-p14ARF / p16INK4a	A300-340A	Bethyl Laboratories
Mouse monoclonal anti-β-Actin (clone AC-15)	A5441	Sigma-Aldrich

Supplementary Table S4. List of antibodies used for immunoblotting in this study.

Gene name (alternative name[s])	Oligo orientation	Sequence (5' – 3')
MAP7 (Ensconsin/E-MAP-115)	Forward	TCATCATGCCCTACAAAGCTG
MAP7 (Ensconsin/E-MAP-115)	Reverse	TGCCAGATGTGAGGAAGAGTA
LATS2	Forward	GCCTTGAGGGAAATCAGATATTCC
LATS2	Reverse	GGGGAGGTCTGCTTAATGACCC
NUSAP1	Forward	GCCAAGAGTCTGGGTCTCCG
NUSAP1	Reverse	GCTGTGAGTCAGGGTCCACA
CENPA	Forward	TTCCTCCCATCAACACAGTCG
CENPA	Reverse	CACACCACGAGTGAATTTAACAC
CENPF	Forward	CAAGAATATGCACAACGTCCTGC
CENPF	Reverse	GAACGCCTGTTCAGCTCTG
CENPM	Forward	GAACACGGCCACCATCTTGC
CENPM	Reverse	GAAGCTGGCATCCACATGGC
KIFC2 (MCAK)	Forward	GCTGAGGCCAGGGACATCTT
KIFC2 (MCAK)	Reverse	GTGCCTGTCTGGCCATAGGT
SPC25	Forward	GCAGAGAGGTTGAAAAGGCTGC
SPC25	Reverse	TCTGCTAGGCCCTCAAGATGA
SPDL1 (CCDC99/Spindly)	Forward	GAGTTCCCGCTGACGCTGAG
SPDL1 (CCDC99/Spindly)	Reverse	TGAGGGCACTGGGTCTCTGG
PDS5B	Forward	AGGAGAGCACAGCAGAGAGC
PDS5B	Reverse	AGCTGCTTGTTTGGAGCGTC
STAG2 (SA2)	Forward	CAAGCATGACCGAGATATAGCAC
STAG2 (SA2)	Reverse	CCGTACTAACACGCCAATGAAT
TPX2	Forward	GAGATGTGCCACTCCTGTAATC
TPX2	Reverse	CTTGGCTTTCTCTGGGGAAG
KIF11 (Eg5)	Forward	TGTTTGATGATCCCCGTAACAAG
KIF11 (Eg5)	Reverse	CTGAGTGGGAACGACTAGAGT
AURKA	Forward	GGAATATGCACCACTTGGAACA
AURKA	Reverse	TAAGACAGGGCATTTGCCAAT
TBP	Forward	GGAGAGTTCTGGGATTGTAC
TBP	Reverse	CTTATCCTCATGATTACCGCAG

Supplementary Table S5. List of oligonucleotides used for quantitative PCR in this study.
